# Man-Specific Lectins from Plants, Fungi, Algae and Cyanobacteria, as Potential Blockers for SARS-CoV, MERS-CoV and SARS-CoV-2 (COVID-19) Coronaviruses: Biomedical Perspectives

**DOI:** 10.3390/cells10071619

**Published:** 2021-06-28

**Authors:** Annick Barre, Els J. M. Van Damme, Mathias Simplicien, Sophie Le Poder, Bernard Klonjkowski, Hervé Benoist, David Peyrade, Pierre Rougé

**Affiliations:** 1 UMR 152 PharmaDev, Institut de Recherche et Développement, Faculté de Pharmacie, Université Paul Sabatier, F-31062 Toulouse, France; annick.barre@univ-tlse3.fr (A.B.); simplicien.mathias@gmail.com (M.S.); herve.benoist@ird.fr (H.B.); 2 Department of Biotechnology, Faculty of Bioscience Engineering, Ghent University, B-9000 Ghent, Belgium; elsjm.vandamme@ugent.be; 3 UMR Virologie, INRA, ANSES, Ecole Nationale Vétérinaire d’Alfort, F-94700 Maisons-Alfort, France; sophie.lepoder@vet-alfort.fr (S.L.P.); bklonjkowski@vet-alfort.fr (B.K.); 4 UMR5129 Laboratoire des Technologies de la Microélectonique, Université Grenoble Alpes, CNRS, CEA-Leti Minatec, CEDEX, F-38054 Grenoble, France; david.peyrade@cea.fr

**Keywords:** plant lectins, mannose-specific lectins, plant lectins, algae lectins, fungi lectins, cyanobacteria lectins, pea lectin, artocarpin, snowdrop lectin, biomedical applications, coronaviruses, SARS-CoV, MERS-CoV, SARS-CoV-2, COVID-19

## Abstract

Betacoronaviruses, responsible for the “Severe Acute Respiratory Syndrome” (SARS) and the “Middle East Respiratory Syndrome” (MERS), use the spikes protruding from the virion envelope to attach and subsequently infect the host cells. The coronavirus spike (S) proteins contain receptor binding domains (RBD), allowing the specific recognition of either the dipeptidyl peptidase CD23 (MERS-CoV) or the angiotensin-converting enzyme ACE2 (SARS-Cov, SARS-CoV-2) host cell receptors. The heavily glycosylated S protein includes both complex and high-mannose type *N*-glycans that are well exposed at the surface of the spikes. A detailed analysis of the carbohydrate-binding specificity of mannose-binding lectins from plants, algae, fungi, and bacteria, revealed that, depending on their origin, they preferentially recognize either complex type *N*-glycans, or high-mannose type *N*-glycans. Since both complex and high-mannose glycans substantially decorate the S proteins, mannose-specific lectins are potentially useful glycan probes for targeting the SARS-CoV, MERS-CoV, and SARS-CoV-2 virions. Mannose-binding legume lectins, like pea lectin, and monocot mannose-binding lectins, like snowdrop lectin or the algal lectin griffithsin, which specifically recognize complex *N*-glycans and high-mannose glycans, respectively, are particularly adapted for targeting coronaviruses. The biomedical prospects of targeting coronaviruses with mannose-specific lectins are wide-ranging including detection, immobilization, prevention, and control of coronavirus infection.

## 1. Introduction

**Figure 1:**
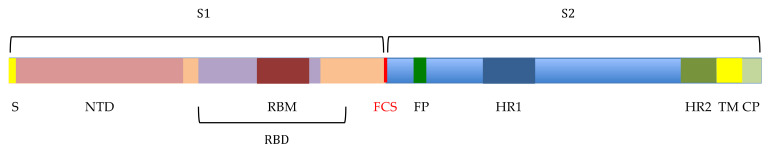
Molecular organization of the SARS-CoV-2 S-glycoprotein, made up of two linked S1 and S2 subunits that are further separated at the furin cleavage site (FCS). The S1 subunit consists of a signal peptide (S), followed by the *N*-terminal domain (NTD) containing the receptor-binding domain (RBD) interacting with the ACE2 receptor via the RBM motif (RBM). The S2 subunit comprises the fusion peptide (FP), two heptad repeats HR1 and HR2, the transmembrane spanning domain (TM) that anchors the S protein into the virion membrane, and the cytoplasmic domain CP.

The human Betacoronaviruses SARS-CoV, MERS-CoV, and SARS-CoV-2 responsible for the pandemic COVID 19 that still rages worldwide, share common structural characteristics that account for their transmissibility and infectivity among sensitive people. The virions are covered by spikes, which protrude outside of the lipid bilayer forming the envelope of coronaviruses [1,2,3]. Spikes are built up from the homotrimeric association of the S protein), an acidic class I viral fusion glycoprotein of ~130 kDa which consists of two S1 and S2 subunits [4,5,6,7]. The S protein is cleaved between the S1 and S2 subunits (S1/S2 cleavage site) and at an additional site S2’ by host cell protease. The S1 subunit contains a receptor-binding domain (RBD), which allows the virus to specifically recognize host cell receptors via a receptor-binding motif (RBM). The dipeptidyl peptidase 4 (DPP4), also known as CD26, was identified as the host receptor for MERS-CoV [8], whereas the angiotensin-converting enzyme 2 (ACE2) serves as the host receptor for SARS-CoV and SARS-CoV-2 [9]. The S2 subunit is subsequently involved in the fusion of both coronavirus and host cell membranes through the fusion peptide (FP) and the first heptad repeat HR1 [10], allowing the entry of the viral RNA genome into the host cell, which will further trigger the replication of the coronavirus at the detriment of the host cell (Figure 1).

The S proteins forming the coronavirus spikes, consist of heavily *N*-glycosylated proteins containing a high number of potential *N*-glycosylation sites NXT/S, actually occupied by complex-type glycans, high-mannose type glycans, and a few hybrid type glycans: 22 sites for SARS-CoV and SARS-CoV-2, and 23 sites for MERS-CoV, respectively [11,12]. A very limited number of potential *O*-glycosylation sites occupied by GalNAc and T/Tn-antigens have been identified, especially in the SARS-CoV-2 S-glycoprotein [12]. According to their heavily *N*-glycosylated character, S-glycoproteins of coronaviruses represent particularly relevant targets for mannose-specific (Man-specific) lectins, a heterogeneous group of proteins involved in the recognition of Man, mannosides, and complex Man-containing glycans, already known for their potential anti-viral and anti-cancer properties [13,14,15,16,17,18].

The Man-specific lectins are widely distributed in all the living organisms since they have been isolated and characterized from viruses, bacteria, fungi, algae, plants, animals, and humans [19]. Even though they constitute a disparate group of proteins poorly related by their phylogenetic relationship and structural features, they share a very common property to specifically recognize Man and their derivatives, including mannosides, oligomannosides, and high-mannose glycans [20]. This specific recognition also concerns other Man-containing glycans like the complex *N*-glycans which are built around a trimannosyl Manα1,3Manα1,6Man core, readily recognized by Man-specific legume lectins [21,22]. As shown for plant lectins, the differences observed in the specific recognition of Man-containing glycans, essentially depend on both the axial position of the hydroxyl group at C2 in Man which is more or less strictly recognized by the carbohydrate-binding sites (CBSs) of lectins, and the type of glycosidic bonds α1,2, α1,3 or α1,6, and their internal or external position in the glycan chain, recognized by lectins [23]. According to these discrepancies and with respect to the diversity of *N*-glycans occurring at the surface of the coronavirus spikes, many distinct Man-specific lectins from plants, algae, fungi, and bacteria, could serve as relevant glycan probes for SARS-CoV, MERS-CoV, and SARS-CoV-2 viruses.

In addition to their Man-binding capacity, Man-specific lectins from plants, algae, fungi, and bacteria, have been largely studied with respect to their anti-viral properties against different types of enveloped viruses, including HIV-1 [24], papilloma virus [25], herpes virus, hepatitis C virus [26], and Ebola virus [27]. In this respect, the algal lectin griffithsin [28], the cyanobacteria lectins cyanovirin [29], actinohivin [30], and microvirin [31], and various GNA-related lectins like NPA from daffodil (*Narcissus pseudonarcissus*) and ASA from garlic (*Allium sativum*) [32,33,34], have been particularly well documented. Most of these Man-specific lectins prevent the virus replication, at least under in vitro conditions, by interfering with the Man-containing *N*-glycans present on the cell surface of the virion envelope [35]. Depending on the virus, different Man-containing glycans serve as targets for the Man-specific lectins, e.g., gp120 for HIV-1 [36] or hemagglutinin for influenza virus [37]. Owing to their smaller size, compared to other plant Man-specific lectins, Man-specific lectins from cyanobacteria such as CVN-N (cyanovirin), MVN (microvirin), and AH (actinohivin) from actinobacteria, have been deeply investigated for their anti-viral properties. In addition, only very few publications report the ability of Man-specific lectins from higher plants to interact with glycans of the coronavirus envelope [13,38,39,40].

The present review aims at presenting a detailed analysis of the Man-specific lectins from plants, algae, fungi, and bacteria that could serve as relevant probes for targeting the *N*-glycans coating the spikes of SARS-CoV, MERS-CoV, and SARS-CoV-2 coronaviruses, with a focus on their potential biomedical applications in controlling coronavirus infections, notably the COVID-19 pandemic associated with SARS-CoV-2.

## 2. Man-Specific Lectins from Higher Plants, Fungi, Algae, and Cyanobacteria

Mannose-specific lectins consist of a group of lectins that are widely distributed in all living organisms and, especially, in higher plants (monocot and dicot plants), in fungi (Ascomycota and Basidiomycota), in the different groups of algae (essentially red and green algae), and in bacteria, mainly the cyanobacteria (formerly known as blue algae). Although they belong to quite different structural scaffolds [20], all of these lectins contain CBSs that display a more or less strict specificity for mannose (Man) and Man-containing glycoproteins.

### 2.1. Man-Specific Lectins from Higher Plants

**Figure 2:**
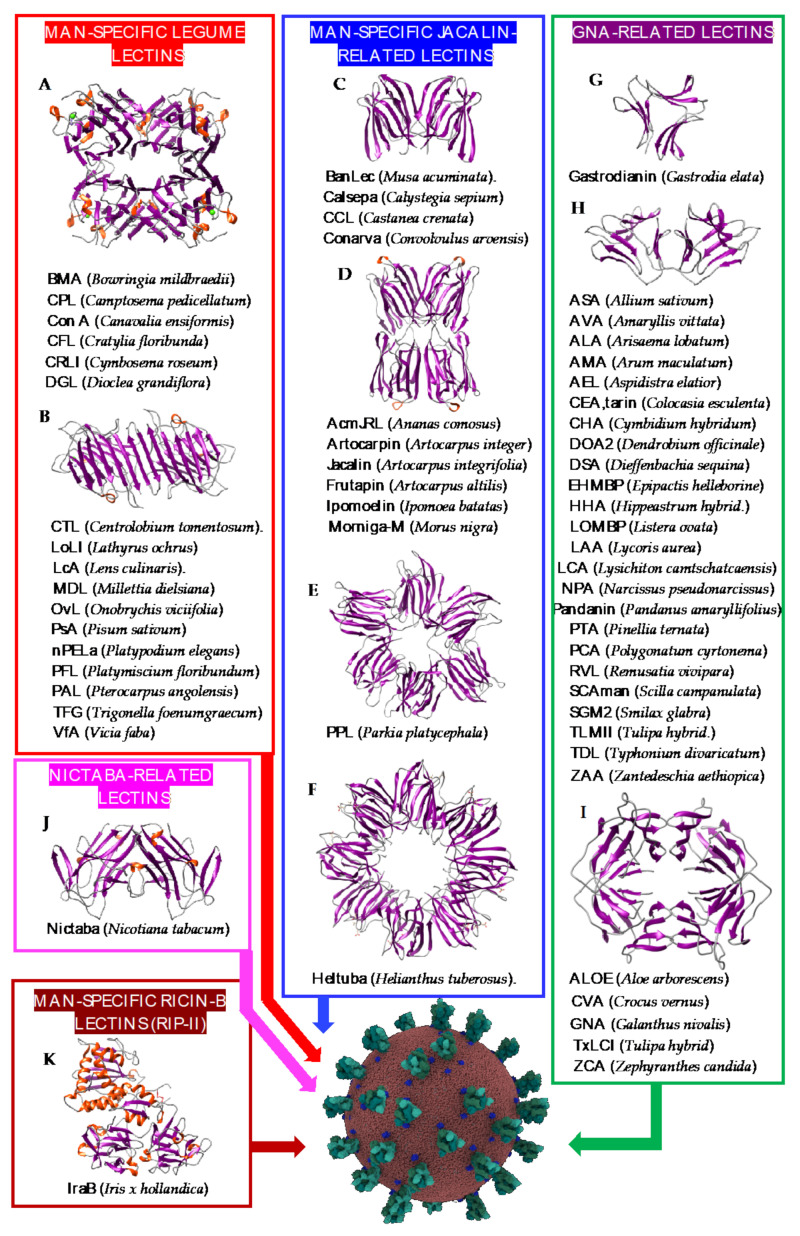
Overview of lectins from representative genera/species belonging to higher plant families, with their associated structural scaffolds, able to recognize the spike proteins from coronaviruses (*Coronavirus* Credit: Maria Voigt/RCSB PDB). A-K correspond to lectins used as structural scaffolds ((**A**): Con A, (**B**): LcA, (**C**): BanLec, (**D**): Morniga-M, (**E**): PPL, (**F**): Heltuba, (**G**): Gastrodianin, (**H**): ASA, (**I**): GNA, (**J**): Nictaba, (**K**): IraB. References [41,42,43,44,45,46,47,48,49,50,51,52,53,54,55,56,57,58,59,60,61,62,63,64,65,66,67,68,69,70,71,72,73,74,75,76,77,78,79,80,81,82,83,84,85,86,87,88,89,90,91,92,93,94,95,96,97,98] correspond to the lectins presented in the figure.

Seeds and tubers of higher plants often contain Man-specific lectins which have been associated with different structural scaffolds (Figure 2):

- The β-sandwich structure (jelly-roll structural scaffold), present in legume lectins. They are classically divided into two groups of single- and two-chain lectins, according to the cleavage (two-chain) or the absence of cleavage (single-chain) of their protomers. Single-chain, tetravalent lectins consist of the non-covalent association of four identical protomers (α_4_), each containing a CBS that specifically recognizes Man and its derivatives. In two-chain lectins, a proteolytic cleavage of the protomeric chain results in the separation of a light (α-chain) and a heavy (β-chain), which remain associated with non-covalent bonds. Two-chain, bivalent lectins (α_2_β_2)_) result from the non-covalent association of two identical two-chain protomers, each possessing a CBS specific for Man and its derivatives.

Both Con A (concanavalin A) from jack bean seeds (*Canavalia ensiformis*) (Figure 2A) and LcA (lentil lectin) from *Lens culinaris* (Figure 2B) are classical examples of single-chain and two-chain lectins, respectively.

- The β-prism I structure (β-barrel structural scaffold), present in the jacalin-related lectin group. Their protomers are organized in three bundles of four antiparallel β-strands arranged into a β-prism structure along a longitudinal axis. Depending on the number of identical protomers associated by non-covalent bonds, Man-specific jacalin-related lectins consist of bivalent (two CBSs) lectins, e.g., Calsepa from hedge bindweed *Calystegia sepium* [61] (Figure 2C); tetravalent (four CBSs) lectins, e.g., Artocarpin from black mulberry *Artocarpus integer* [65] (Figure 2D); hexavalent (six CBSs) lectins, e.g., PPL from the African locust bean *Parkia platycephala* [70] (Figure 2E); or octavalent (eight CBSs) lectins, e.g., Heltuba from the Jerusalem artichoke *Helianthus tuberosus* [71] (Figure 2F).
- The β-prism II structure (β-trefoil structural scaffold), present in the GNA-related lectin group. The protomer consists of three bundles of four antiparallel β-strands arranged into a flattened β-trefoil structure around a central pseudoaxis. A CBS occurs in each of the bundles of β-strands. Except for gastrodianin, which is composed of a single protomer (Figure 2G), lectins belonging to the GNA-related group result from the non-covalent association of two protomers, e.g., the hexavalent NPA from daffodil (*Narcissus pseudonarcissus*) bulbs [80] (Figure 2H) or four protomers, e.g., the dodecavalent GNA from snowdrop (*Galanthus nivalis*) bulbs [94] (Figure 2I).
- Two covalently linked β-trefoil-domains form the B-chain of the lectin from *Iris hollandica*, a type II ribosome-inactivating protein (RIP-II) (Figure 2J). Each domain contains a CBS which can accommodate Man and Gal/GalNAc. This Man-binding property confers an unusual Man-specificity to the Iris RIP-II, which readily differs from the Gal/GalNAc-specificity of other RIP-IIs [97].
- Nictaba, the jasmonic acid-induced tobacco (*Nicotiana tabacum*) lectin displays specificity for chitin and GlcNAc oligomers but it can also bind to high mannose glycans with a lower affinity, as shown from glycan array experiments. The molecular structure of Nictaba is still unknown but modeling experiments suggest a β-sandwich structure for this tobacco lectin [98] (Figure 2K).

**Figure 3:**
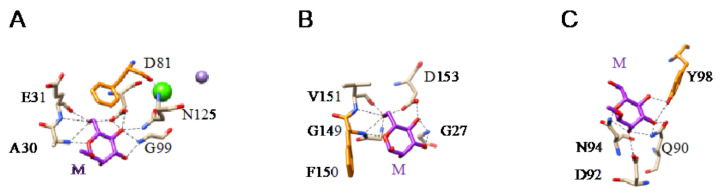
(**A**). Network of hydrogen bonds (black dashed lines) anchoring Man (M) to the amino acid residues A30, E31, D81, G99, and N135, forming the CBS of LoLI from *Lathyrus ochrus* (PDB code 1LOB). Aromatic residue F123 participating in stacking interaction with the pyranose ring of Man is colored orange. (**B**). Network of hydrogen bonds (black dashed lines) anchoring Man (M) to the amino acid residues G27, G149, V151, and D153, forming the CBS of Morniga-M from *Morus nigra* (PDB code 1XXR). Aromatic residue F150 involved in stacking interaction with the pyranose ring of Man is colored orange. (**C**). Network of hydrogen bonds (black dashed lines) anchoring Man (M) to the amino acid residues Q90, D92, and N94, forming the CBS of PCL from *Polygonatum cyrtonema* (PDB code 3A0D). Aromatic residue Y98 participating in stacking interaction with the pyranose ring of Man is colored orange.

**Figure 4:**
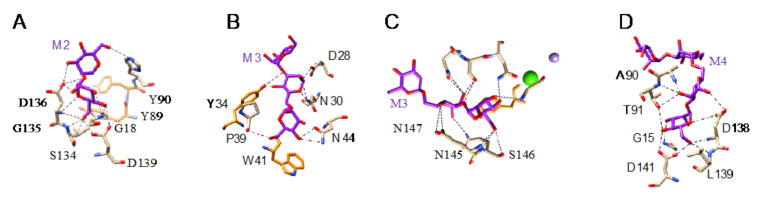
(**A**). Network of hydrogen bonds (black dashed lines) anchoring Manα1,2Man (M2) to Heltuba from *Helianthus tuberosus* (PDB code 1C3N). Aromatic residues Y89 and Y90 participating in stacking interactions with the pyranose rings of mannobiose are colored orange. (**B**). Network of hydrogen bonds (black dashed lines) anchoring Manα1,6Manα1,3Man (M3) to GNA from *Galanthus nivalis* (PDB code 1JPC). Aromatic residues Y89 and Y90 participating in stacking interactions with the pyranose rings of mannobiose are colored orange. (**C**). Network of hydrogen bonds (black dashed lines) anchoring Manα1,3Manα1,6Man (M3) to PAL from *Pterocarpus angolensis* (PDB code 1Q8V). Aromatic residues Y89 and Y90 participating in stacking interactions with the pyranose rings of mannobiose are colored orange. (**D**). Network of hydrogen bonds (black dashed lines) anchoring mannotetraose (M4) to artocarpin from *Artocarpus integer* (PDB code 1VBP).

All of these lectins readily interact with Man and oligomannosides but, more interestingly, they display a higher affinity for more complex Man-containing glycan chains that are usually associated with cell surfaces. In this respect, different Man-specific lectins have been co-crystallized in the presence of Man (Figure 3A–C), di-mannoside (Figure 4A), tri-mannoside core Manα1,3Manα1,6Man (Figure 4B,C), and tetra-mannoside (Figure 4D) occurring in *N*-glycans of the complex and high-mannose type.

**Figure 5:**
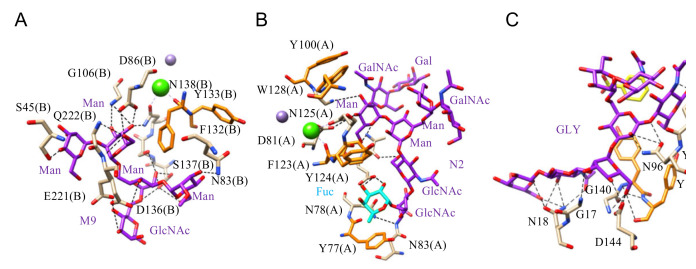
(**A**). Network of hydrogen bonds (black dashed lines) anchoring Man9 (M9) to PAL, the *Pterocarpus angolensis lectin* (PDB code 2PHN). Aromatic residues F132 and Y133 participating in stacking interactions with the pyranose rings of the oligomannoside are colored orange. (**B**). Network of hydrogen bonds (black dashed lines) anchoring N2 oligosaccharide to LoLII, isolectin II from *Lathyrus ochrus* (PDB code 1LGB). Aromatic residues Y77, Y100, F123, Y124, and W128 involved in stacking interactions with the pyranose rings of the oligosaccharide, are colored orange. The α1,6-linked fucose (Fuc) participates in the H-bond network. (**C**). Network of hydrogen bonds (black dashed lines) anchoring a biantennary glycan (GLY) to Calsepa from *Calystegia sepium* (PDB code 5XFI). Aromatic residue Y141 and Y142 participating in stacking interaction with the pyranose rings of the glycan are colored orange.

In addition, results from glycan arrays and binding assays, and X-ray crystallography of lectins co-crystallized in the presence of complex glycan chains revealed that Man-binding lectins from higher plants also accommodate more complex *N*-glycan chains, through a complex network of hydrogen bonds and stacking interactions between the pyranose ring of sugars and aromatic residues (F, W, Y) located in the vicinity of the CBS of lectins (Figure 5A–C).

**Figure 6:**
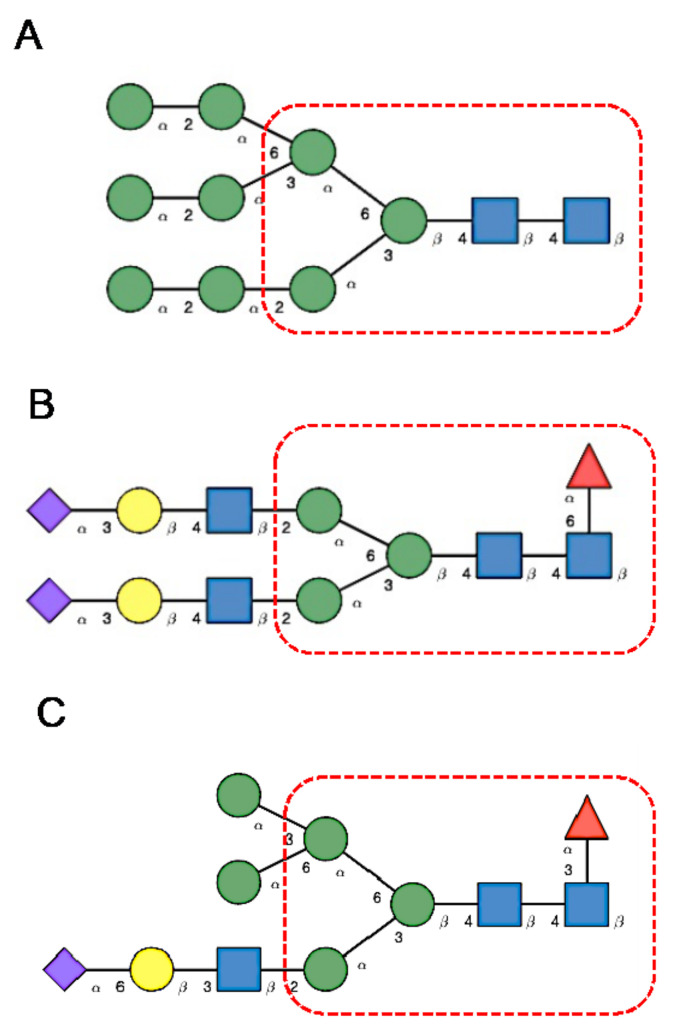
(**A**). High-mannose *N*-glycan, in which only Man (green circles) residues extend the core. (**B**). Complex *N*-glycans with two antennae initiated by a GlcNAc residue (blue square) extending the core. (**C**). Hybrid *N*-glycan with Man extending the Man1,6 arm of the core and GlcNAc extending the Man1,3 arm of the core. The core is delineated by a dashed red line.

**Figure 7:**
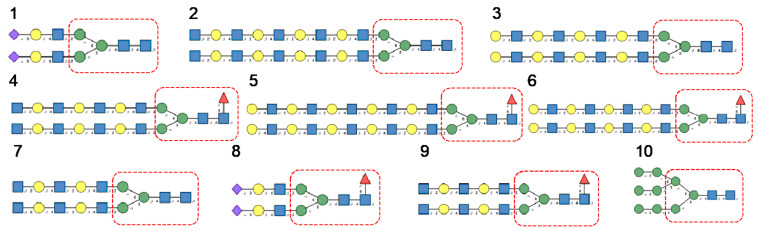
Glycans recognized by Con A in glycan array experiments, arranged from 1 to 10 in decreasing order of affinity.

**Figure 8:**
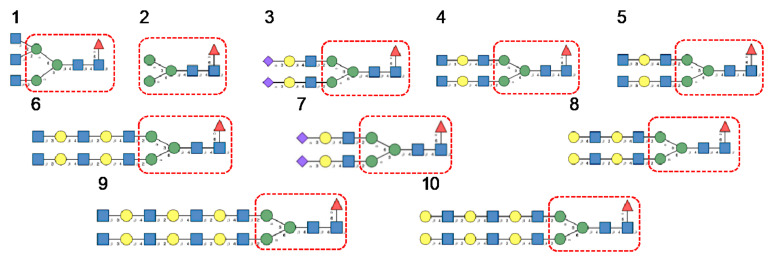
Glycans recognized by PsA in glycan array experiments, arranged from 1 to 10 in decreasing order of affinity.

**Figure 9:**
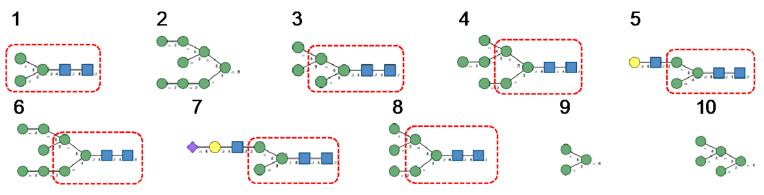
Glycans recognized by GNA in glycan array experiments, arranged from 1 to 10 in decreasing order of affinity.

**Figure 10:**
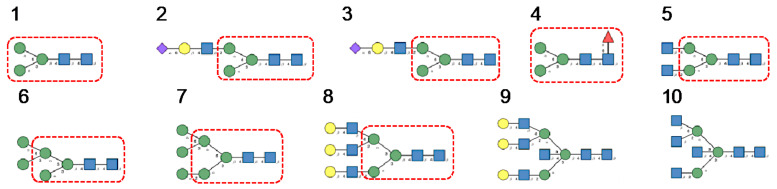
Glycans recognized by Morniga-M in glycan array experiments, arranged from 1 to 10 in decreasing order of affinity.

*N*-glycans are classified into three types, including oligomannose or high-mannose glycans, complex glycans, and hybrid glycans, according to the extensions of their common core sequence Manα1,3(Manα1,6)Manβ1,4GlcNAcβ1,4GlcNAcβ1-Asn (Figure 6A–C):

Glycan array analyses were performed by the Consortium for Functional Glycomics (http://www.functionalglycomics.org, accessed on 14 March 2021) for Con A, a typical member of the Man-specific single-chain lectins from the Fabaceae. Con A yielded the best interaction with the following glycans arranged in decreasing order of affinity (Figure 7):

Glycan array analyses performed for PsA, a typical member of the Man-specific two-chain lectins from the Vicieae (a tribe of Leguminosae/Fabaceae), yielded the best results with the following glycans arranged in decreasing order of affinity (Figure 8):

Glycan array results obtained for GNA, the Man-specific lectin from snowdrop, the prototype of monocot Man-binding lectins, are in decreasing order of affinity (Figure 9):

Results obtained with Morniga-M, a typical representative of the jacalin-related Man-binding lectins, reveal some reactivity towards bisected hybrid glycans (Figure 10):

From the glycan array results and X-ray crystallographic analysis of lectins in complex with Man-containing glycans, it clearly appears that beyond a similar affinity for the trimannosyl Manα1,6Manα1,3Man core occurring in both *N*-glycans of the complex, high-mannose and hybrid types, Man-specific lectins from higher plants are not equivalent and readily differ in their capacity to specifically recognize subtle chemical arrangements in the complex glycan chains. In this respect, Man-specific legume lectins essentially recognize complex glycans α1,6 fucosylated at the GlcNAc residue linked to asparagine, whereas Man-specific lectins belonging to the GNA-related lectin group, essentially recognize more or less branched high-mannose *N*-glycans. The Man-specific lectins of the jacalin-related group differ from the other lectins by their strong specificity for high-mannose glycans. Depending on the differences observed in the fine specificity towards complex glycans, high-mannose glycans, and hybrid glycans, Man-specific lectins from diverse origins can be used as glycan probes for targeting diverse types of *N*-glycans covering both host cell and viral surfaces.

### 2.2. Man-Specific Lectins from Lower Plants and Fungi

**Figure 11:**
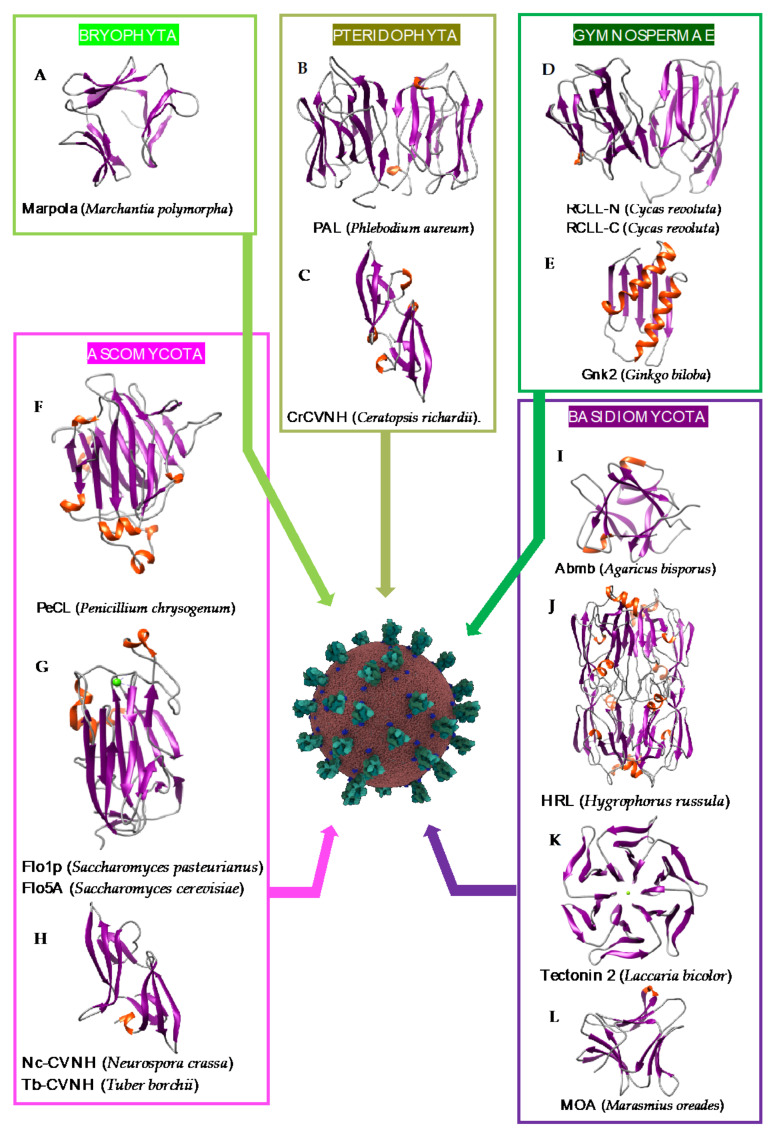
Overview of lectins from the major representative genera/species of lower plants and fungi, with their associated structural scaffolds, able to recognize the spike proteins from coronaviruses (*Coronavirus* Credit: Maria Voigt/RCSB PDB). A-L correspond to lectins used as structural scaffolds (**A**): Marpola, (**B**): PAL, (**C**): CrCVNH, (**D**): RCLL-N, (**E**): Gnk2, (**F**): PeCL, (**G**): Flo1p, (**H**): TbCVNH, (**I**): Abmb, (**J**): HRL, (**K**): Tectonin 2, (**L**): MOA. References [99,100,101,102,103,104,105,106,107,108,109,110,111] correspond to the lectins presented in the figure.

**Figure 12:**
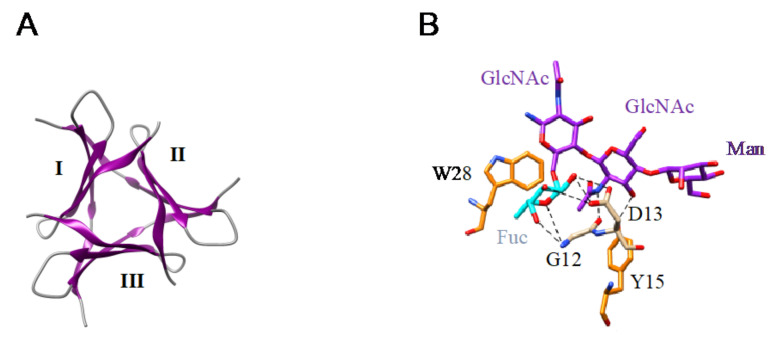
(**A**). Arrangement of the three antiparallel β-sheets (I, II, III) forming the PhosL homotrimer (PDB code 5XZK) to mimic a pseudo-β-trefoil structure. (**B**). Network of hydrogen bonds (black dashed lines) anchoring the fucose (Fuc, colored cyan) α1,6-linked for the first GlcNAc from a fucosylated *N*-glycan to the CBS of PhosL (PDB code 6FX1). Aromatic residues Y89 and Y90 participating in stacking interactions with the pyranose rings of mannobiose are colored orange.

Although they are less documented compared to the lectins from higher plants, a few Man-specific lectins have also been identified in lower plants including Bryophyta, Pteridophyta, and Gymnospermae. Man-specific lectins also occur in both groups of fungi, Ascomycota and Basidiomycota. They usually consist of structural scaffolds very similar to those found in higher plant lectins but few of them also exhibit quite unusual structural scaffolds like the β-propeller fold found in fungi or the α + β fold found in the Gymnospermae (Figure 11A–L):

- The β-sandwich structure (jelly-roll structural scaffold) typical for legume lectins, also occurs in the Man-specific lectin PeCL from *Penicillium chrysogenum*, and in floculins Flo1p from the yeast *Saccharomyces pasteurianum* and Flo5p from *Saccharomyces cerevisiae*.
- The β-prism II structure (β-trefoil structural scaffold), that builds the GNA-related lectin protomer, also occurs in Marpola, the Man-specific lectin isolated from the liverwort *Marchantia polymorpha*, and in mushrooms (Abmb from *Agaricus bisporus*, MOA from *Marasmius oreades*). Special mention should be made to the apparent β-trefoil structure that was found in the mini-lectin PhosL from the mushroom *Pholiota squarrosa*. In fact, PhosL results from the trimeric association of three antiparallel β-sheets, each possessing a CBS, that mimics a β-trefoil structure with three identical CBSs. This newly identified lectin fold is involved in the specific recognition of the core α1,6-fucosylation of fucosylated *N*-glycans [100] (Figure 12A,B).

- A rather unusual 6-bladded β-propeller organization was identified in tectonin 2, the Man-specific lectin from the basidiomycete *Laccaria bicolor* [109]. As commonly observed in other lectins exhibiting a β-propeller structure, tectonin 2 contains 6 identical Man-specific CBSs located at the top of each of the six blades forming the β-propeller.
- Another quite unusual structural scaffold, which consists of an antiparallel five-stranded β-sheet associated to two α-helices (α + β fold), occurs in the monomeric ginkbilobin 2, a Man-specific lectin from *Ginkgo biloba* [103]. Hydrophilic residues occurring on the loops connecting the β-strands associated with charged residues of two β-strands, to form a Man-binding pocket located at the top of the monomer [103].

**Figure 13:**
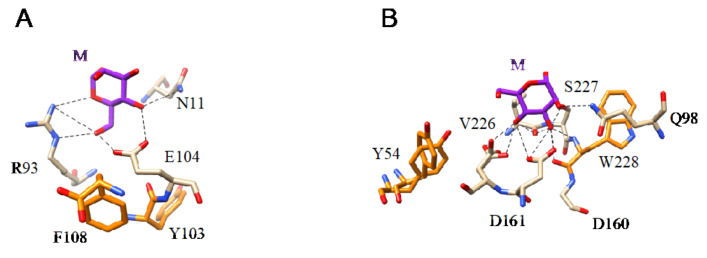
(**A**). Network of hydrogen bonds (black dashed lines) anchoring Man (M) to the amino acid residues N11, R93, and E104, forming the CBS of ginkbilobin from *Ginkgo biloba* (PDB code 4XRE). Aromatic residue Y103 and F108 participating in weak stacking interactions with the pyranose ring of Man are colored orange. (**B**). Network of hydrogen bonds (black dashed lines) anchoring Man (M) to the amino acid residues Q98, D160, D161, V226, and S227, forming the CBS of flocculin Flo5 from *Saccharomyces cerevisiae* (PDB code 2XJP). Aromatic residues Y54 and W228 involved in stacking interactions with the pyranose ring of Man are colored orange.

**Figure 14:**
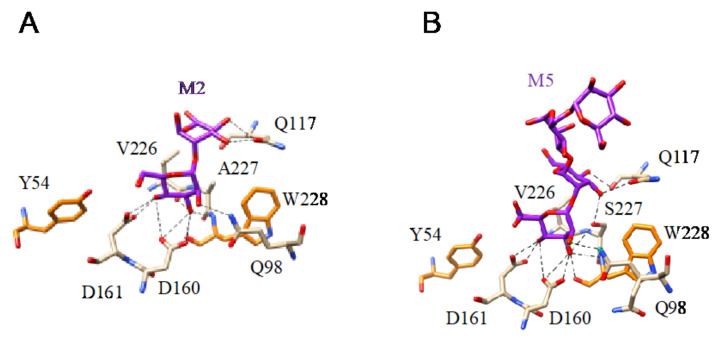
(**A**). Network of hydrogen bonds (black dashed lines) anchoring Mannobiose (M2) to the amino acid residues forming the CBS of flocculin Flo5 from *Saccharomyces cerevisiae* (PDB code 2XJU). Aromatic residue Y54 and FW228 participating in stacking interactions with the pyranose ring of the first Man residue are colored orange. (**B**). Network of hydrogen bonds (black dashed lines) anchoring Mannopentaose (M5) to the amino acid residues forming the CBS of flocculin Flo5 from *Saccharomyces cerevisiae* (PDB code 2XJT). Aromatic residue Y54 and W228 involved in stacking interactions with the pyranose ring of the first Man of the oligomannoside are colored orange.

The mannose-binding specificity of lectins from lower plants (Bryophyta, Pteridophyta, Gymnospermae), yeasts, and mushrooms (Ascomycota and Basidiomycota), is directed against both Man, mannosides, and more complex high-mannose glycans, as shown from the X-ray crystallographic studies for various Man-specific lectins complexed to Man and oligomannosides (Figure 13 and Figure 14).

**Figure 15:**
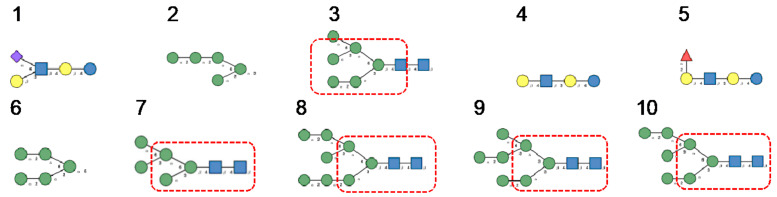
Glycans recognized by TbCVN in glycan array experiments, arranged from 1 to 10 in decreasing order of affinity.

**Figure 16:**
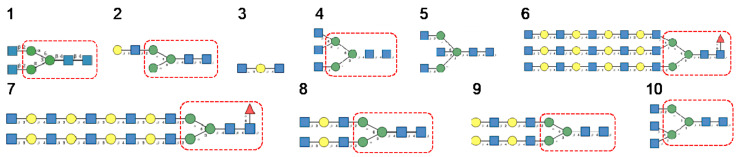
Glycans recognized by ABA in glycan array experiments, arranged from 1 to 10 in decreasing order of affinity.

**Figure 17:**
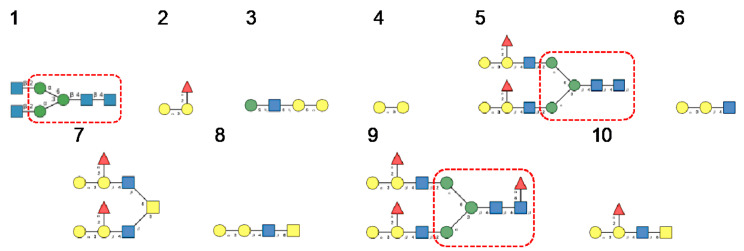
Glycans recognized by MOA in glycan array experiments, arranged from 1 to 10 in decreasing order of affinity.

In the glycan array analyses performed at Consortium for Functional Glycomics for TbCVN, the *Tuber borchii* Man-specific lectin yielded the best results with the following glycans arranged in decreasing order of affinity (Figure 15):

Glycan array studies performed for ABA, the *Agaricus bisporus* lectin, yielded the best results with the following glycans arranged in decreasing order of affinity (Figure 16):

Glycan array analyses performed for the lectin MOA from the *Marasmius oreades* mushroom, yielded the best results with the following glycans arranged in decreasing order of affinity (Figure 17):

Mannose-specific lectins from Ascomycota, such as the TbCVN and NcCVN cyanovirin-related lectins from *Tuber borchii* (TbCVN) and *Neurospora crassa* (NcCVN), usually differ from lectins of the Basidiomycota by a more pronounced affinity to high-mannose glycans and a lesser affinity for complex type glycans, such as ABA from *Agaricus bisporus* and MOA from *Marasmius oreades*.

### 2.3. Man-Specific Lectins from Algae and Bacteria

Seaweeds belonging to the different classes of algae, including red algae (Rhodophyta), brown algae (Phaeophyta), green algae (Chlorophyta), and yellow-green algae (Chrysophyta), are well-known sources of Man-specific lectins, together with the lectins from Actinobacteria and Cyanobacteria, formerly classified as blue algae. 

Seaweed Man-specific lectins exhibit well-identified structural scaffolds but some of them also exhibit unrelated structural scaffolds that still remain to be characterized.

**Figure 18:**
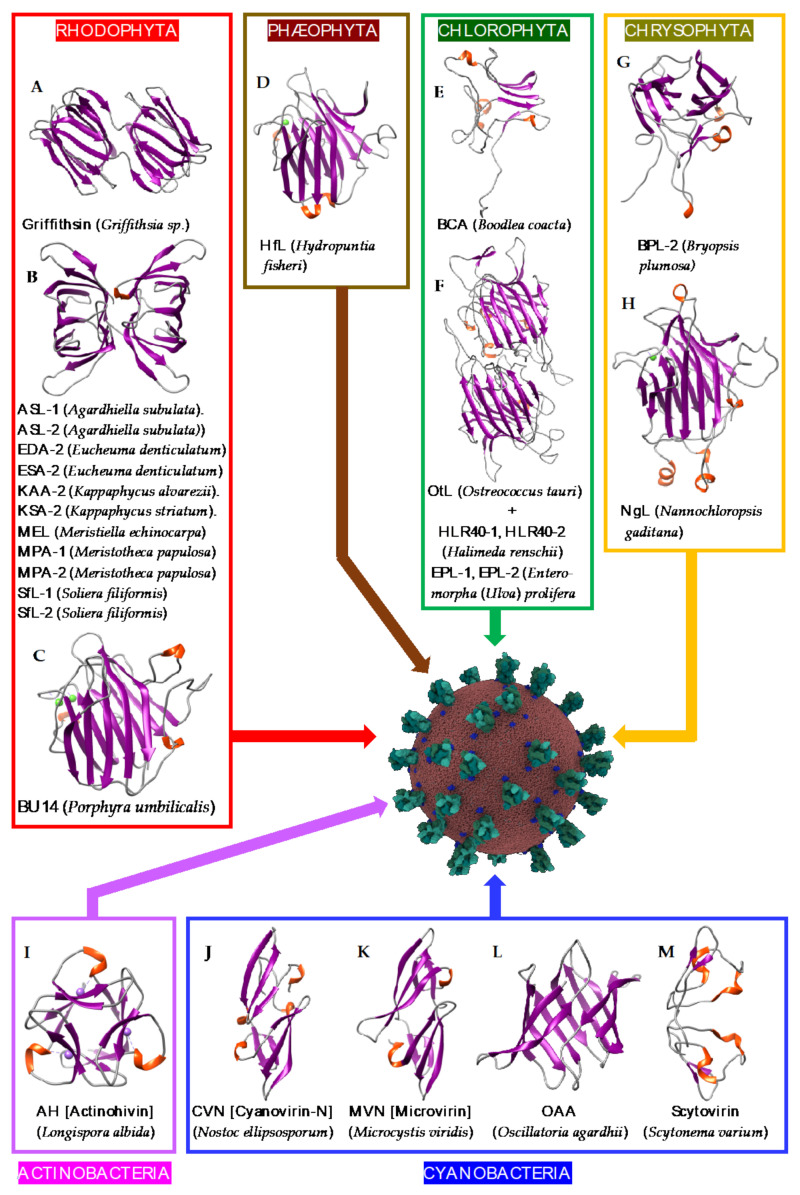
Overview of lectins from representative genera/species of algae and cyanobacteria, with their associated structural scaffolds, able to recognize the spike proteins from coronaviruses (*Coronavirus* Credit: Maria Voigt/RCSB PDB). A-M correspond to lectins used as structural scaffolds ((**A**): Griffithsin, (**B**): ASL-1, (**C**): BU14, (**D**): HfL, (**E**): BCA, (**F**): OtL, (**G**): BPL-2, (**H**): NgL, (**I**): Actinohivin, (**J**): CVN, (**K**): MVN, (**L**): OAA, (**M**): Scytovirin. References [28,29,30,112,113,114,115,116,117,118,119,120,121,122,123,124,125,126,127,128,129] correspond to the lectins presented in the figure.

- The β-prism I or β-barrel scaffold occurs predominantly in red algae (Rhodophyta) but is less common in other classes of algae (Figure 18A–M). Algae of the genera *Agardhiella*, *Eucheuma*, *Kappaphycu*s, *Meristiella*, *Meristotheca,* and *Solieria* exhibit this type of molecular organization, in which the CBS is located at the top of the barrel structure.
- A less common β−prism II organization occurs in red algae (*Grateloupia chiangii*) and green algae (*Boodlea coacta*), in which CBSs are located in the grooves delineated by the bundles of β-strands. The MFP2-like structural scaffold, related to the β-prism II organization, occurs in the green alga *Bryopsis plumosa*.
- A rather uncommon β-sandwich organization was found in the red alga *Hydropuntia* (*Gracilaria*) *fisheri*, and in the green alga *Ostreococcus tauri*.
- It is interesting to note that the structural scaffolds of many other Man-specific lectins, identified in red algae (*Carpopeltis flabellata*, *Gracilaria bursa-pastoris*) and green algae (*Enteromopha prolifera*, *Halimeda renschii*), still remain unknown and are not apparently related to any of the known structural scaffolds reported in algae.

Man-specific lectins from the actinobacteria (actinohivin) and cyanobacteria/ex-blue algae group (cyanovirin CVN, microvirin MVN, scytovirin SVN, and the *Oscillatoria agardhii* agglutinin OAA), exhibit a β-prism II (β-trefoil) structure and a β-prism I (β-barrel) structure, respectively (Figure 18).

**Figure 19:**
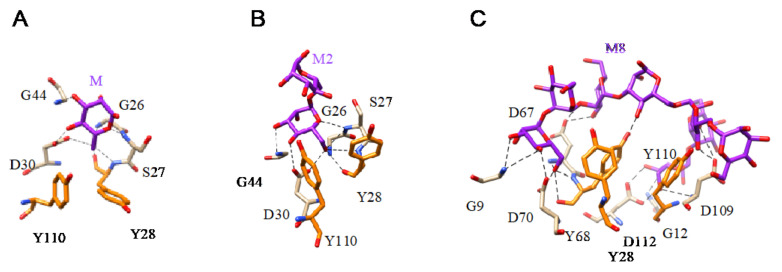
(**A**). Network of hydrogen bonds (black dashed lines) anchoring Man (M) to the amino acid residues forming the CBS of griffithsin (PDB code 2RDK). Aromatic residue Y28 and Y110 participating in stacking interactions with the pyranose ring of Man are colored orange. (**B**). Network of hydrogen bonds (black dashed lines) anchoring 6α-mannobiose (M2) to the amino acid residues of the CBS of griffithsin (PDB code 2HYQ). Aromatic residue Y28 and Y110 involved in stacking interactions with the pyranose ring of the first Man residue are colored orange. (**C**). Network of hydrogen bonds (black dashed lines) anchoring a high-mannose branched glycan (M8) to the amino acid residues of the CBS of griffithsin (PDB code 3LL2). Aromatic residue Y28, Y68, and Y110, participating in stacking interactions with the pyranose rings of Man residues, are colored orange.

**Figure 20:**
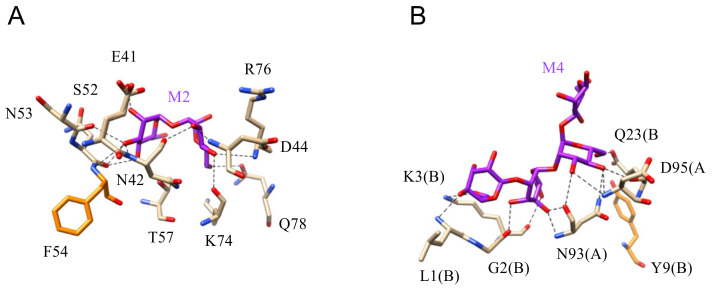
(**A**). Network of hydrogen bonds (black dashed lines) anchoring mannobiose (M2) to the amino acid residues forming the CBS of cyanovirin CVN (PDB code 2PYS). Aromatic residue F54 participating in a very weak stacking interaction with the pyranose ring of Man is colored orange. (**B**). Network of hydrogen bonds (black dashed lines) anchoring mannotetraose (M4) to cyanovirin (PDB code 3GXZ). Amino acid residues of A and B protomers forming the swapped CVN dimer, participate in the interaction with the oligomannoside.

The three-dimensional structures for different bacterial lectins in complex with Man, mannosides, and more complex oligomannoside chains are available at the PDB (Figure 19 and Figure 20).

**Figure 21:**
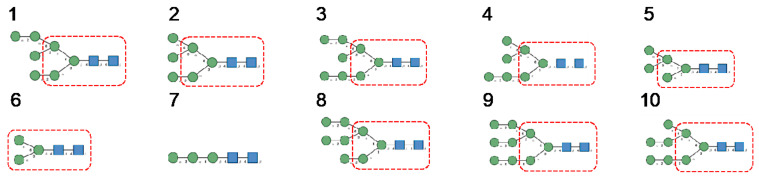
Glycans recognized by ESA-2 in glycan-binding activity measurements, arranged from 1 to 10 in decreasing order of affinity.

**Figure 22:**
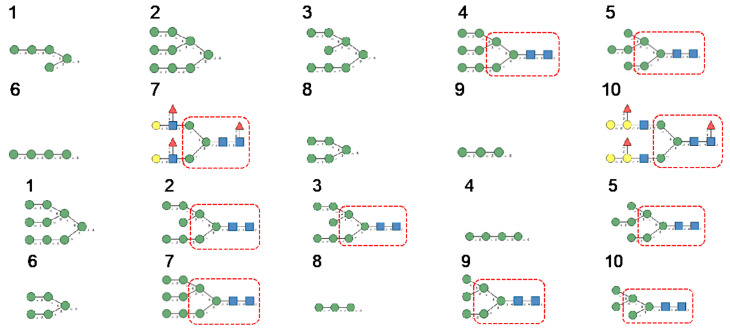
Glycans recognized by CVN-N in glycan array experiments, arranged from 1 to 10 in decreasing order of affinity.

**Figure 23:**
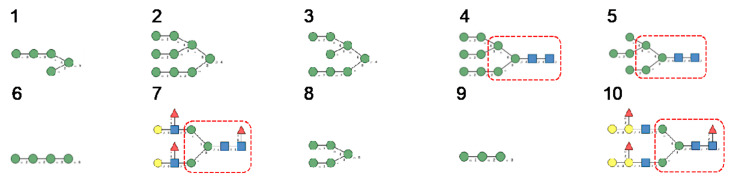
Glycans recognized by actinohivin in glycan array experiments, arranged from 1 to 10 in decreasing order of affinity.

High-mannose glycan-binding activity measured by Hori et al. [130] for ESA-2, the *Eucheuma serra* lectin, yielded the best results with the following glycans arranged in decreasing order of affinity (Figure 21):

Glycan array analyses performed at Consortium for Functional Glycomics for cyanovirin-N CVN-N, a typical member of the cyanobacterial Man-specific lectins, yielded the best results with the following glycans arranged in decreasing order of affinity (Figure 22):

Glycan array studies performed for actinohivin, a typical member of the actinobacteria Man-specific lectins, yielded the best results with the following glycans arranged in decreasing order of affinity (Figure 23):

Man-specific lectins from algae, actinobacteria, and cyanobacteria, almost exclusively interact with high-mannose glycans and, to a very little extent, with complex *N*-glycans. Most of the high-mannose glycans recognized by algal and bacterial lectins readily participate in the glycan equipment of the S proteins forming the spikes that decorate the surface of SARS-CoV, MERS-CoV, and SARS-CoV-2 virions.

## 3. Diversity of Glycans Decorating the Spike-Forming S Glycoproteins of Coronaviruses

**Figure 24:**
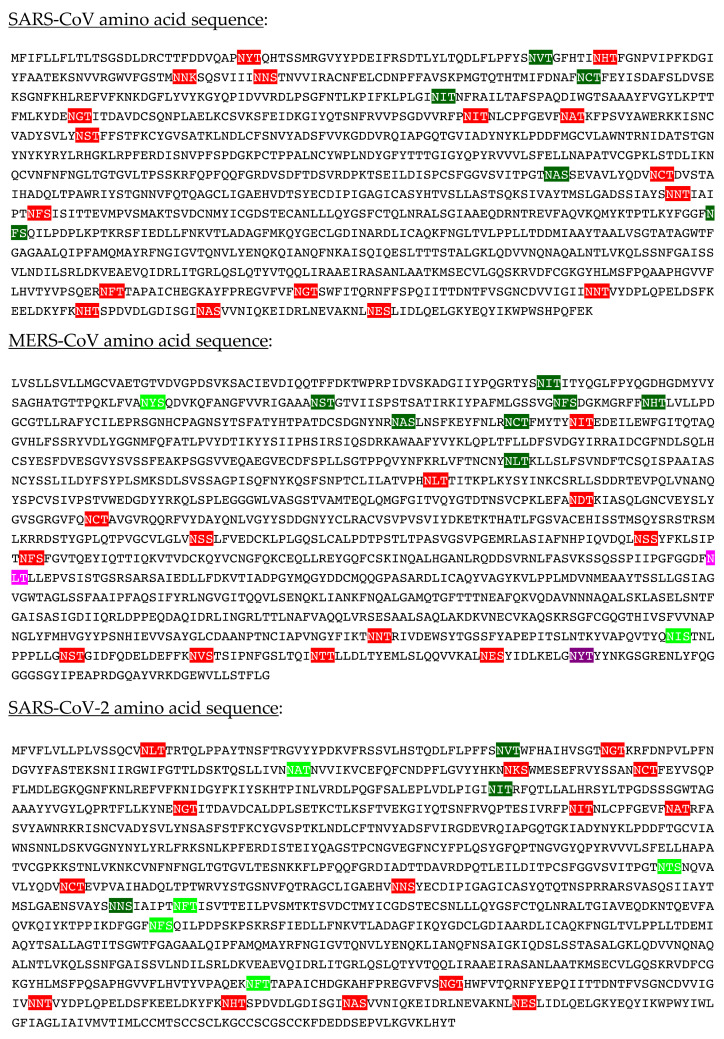
Amino acid sequences of S proteins from SARS-CoV, MERS-CoV, and SARS-CoV-2, showing the *N*-glycosylation sites occupied (almost) exclusively by complex type *N*-glycans (red), high-mannose type *N*-glycans (green), hybrid type *N*-glycans (purple), and predominantly occupied by high-mannose glycans (pale green) or complex glycans (magenta).

The S proteins are organized in homotrimers that form the spikes protruding from the virion lipid bilayer. The S-glycoproteins of SARS-CoV, MERS-CoV, and SARS-CoV-2, consist of heavily *N*-glycosylated proteins consisting of more than 1000 amino-acids, exhibiting a high MW close to 130–140 kDa (133,568 Da for SARS-CoV, 146,594 Da for MERS-CoV, and 141,178 Da for SARS-CoV-2), and an acidic *p*I (5.55 for SARS-CoV, 5.41 for MERS-CoV, and 6.24 for SARS-CoV-2). These proteins contain a high number of potential *N*-glycosylation sites NXT/NXS, that are actually glycosylated (22 for SARS-CoV and SARS-CoV-2, 23 for MERS-CoV) [11,12]. These *N*-glycosylation sites are arrayed along the amino acid sequence of the S-glycoproteins, predominantly at both the *N*- (S1 chain) and *C*-termini (S2 chain) of the polypeptide chain (Figure 24).

**Figure 25:**
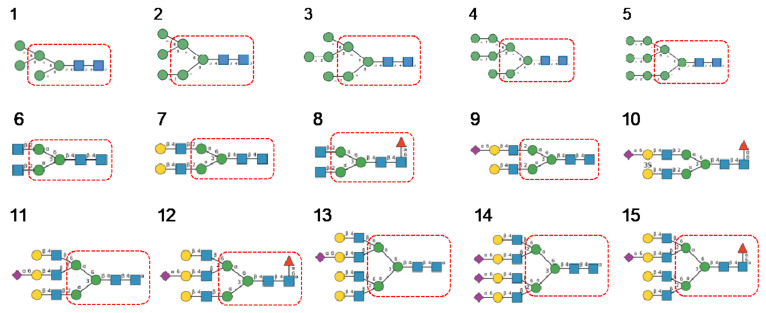
(**1**–**5**). High-mannose-type glycans: GlcNAc_2_Man_5_ (**1**); GlcNAc_2_Man_6_ (**2**), GlcNAc_2_Man_7_ (**3**), GlcNAc_2_Man_8_ (**4**) and GlcNAc_2_Man_9_ (**5**). (**6**–**15**). Complex-type glycans: bi-antennary glycans (**6**,**7**), fucosylated bi-antennary glycan (**8**), sialylated bi-antennary glycan (**9**), sialylated and fucosylated bi-antennary glycan (**10**), sialylated tri-antennary glycan (**11**), sialylated and fucosylated tr-antennary glycan (**12**), sialylated tetra-antennary glycans (**13**,**14**) and sialylated and fucosylated tetra-antennary glycan (**15**).

A detailed study of *N*-glycans present on the S-glycoprotein of coronaviruses revealed the extreme diversity of *N*-glycans covering the coronavirus envelope [11,12]:

- High-mannose type glycans consist essentially of tri-antennary glycans GlcNAc_2_-Man_5–9_ (Figure 25).
- Complex type glycans are represented by essentially fucosylated and often sialylated, bi-, tri-, and tetra-antennary glycans (Figure 25).

- Hybrid type glycans, often bisected, have been detected at different *N*-glycosylation sites but with the exception of the last *C*-terminal *N*-glycosylation site occurring in S-glycoprotein of MERS-CoV which is exclusively occupied by hybrid-type glycans. All other *N*-glycosylation sites are occupied by a mixture of high-mannose, complex, and hybrid glycans (Table 1).
- Scarce potential *O*-glycosylation sites T/S, actually occupied by *O*-glycans, have been identified essentially in the S-glycoprotein of SARS-CoV-2 (T323, S325, and T678) [12].

**Figure 26:**
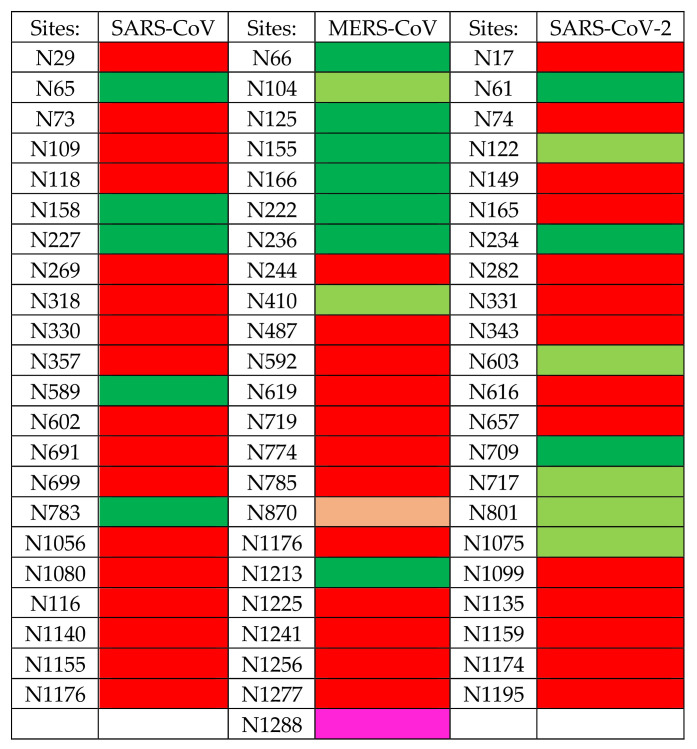
Site-specific *N*-linked glycosylation of SARS-CoV, MERS-CoV, and SARS-CoV-2. Sites containing (almost) exclusively complex glycans (colored red), high-mannose glycans (colored green), and hybrid glycans (colored magenta), are indicated. Sites harboring a mixture of complex glycans, high-mannose glycans, and a few hybrid glycans are colored pale green or pink, depending on the predominant high-mannose glycans (pale green) or complex glycans (orange).

The diversity of *N*-glycosylation occurring at the different *N*-glycosylation sites of both SARS-CoV, MERS-CoV, and SARS-CoV-2, has been summarized in Figure 26. This table suggests that SARS-CoV and SARS-CoV-2 share a rather similar *N*-glycosylation pattern, which readily differs from that observed in MERS-CoV S-glycoprotein. 

In addition to the different *N*-glycosylation patterns found at the glycosylation sites arrayed along the S-glycoprotein trimers forming the coronavirus spikes, spikes from the different coronavirus readily differ by a distinct distribution of the complex and high-mannose glycans at their surface and, especially at the top and the lateral faces of the spikes.

## 4. Structural Organization and Glycosylation Pattern of S Glycoproteins Forming the Spikes of Coronaviruses

**Figure 27:**
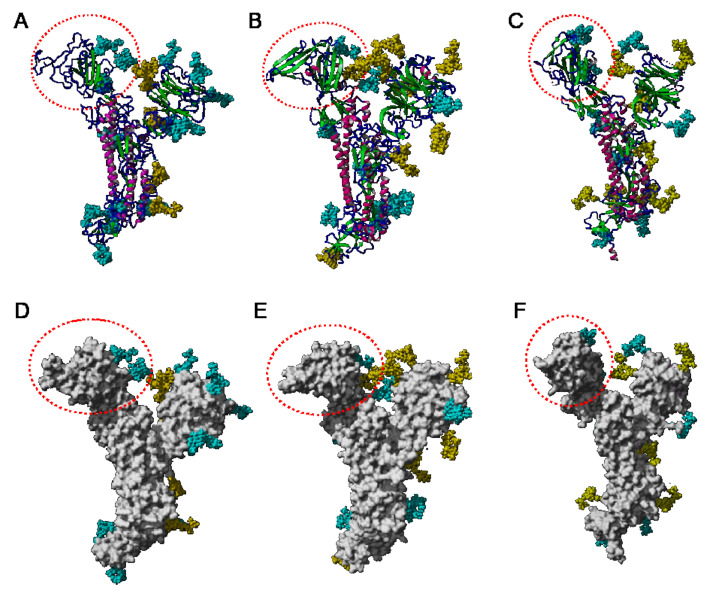
(**A**–**C**). Lateral face of the ribbon diagram representing the heavily glycosylated S glycoprotein of SARS-CoV (PDB code 6ACD) (**A**), MERS-CoV (PDB code 5W9H) (**B**), and SARS-CoV-2 (PDB code 6VXX) (**C**). High-mannose glycans and complex glycans are colored yellow and cyan, respectively. The RBD is indicated by a red dashed circle. (**D**–**F**). Lateral face of the molecular surface (colored grey) of the S-glycoprotein of SARS-CoV (**D**), MERS-CoV (**E**), and SARS-CoV-2 (**F**). High-mannose glycans and complex glycans are colored yellow and cyan, respectively.

The distribution of the high-mannose *N*-glycans and complex *N*-glycans at the surface of the S-glycoprotein strikingly differs depending on the coronaviruses SARS-CoV, MERS-CoV, and SARS-CoV-2 (Figure 27):

- high-mannose glycans are predominantly distributed in the upper part of the S-glycoprotein from MERS-CoV, whereas complex glycans mainly occur along the lower part of the S-glycoprotein.
- complex glycans predominate in the upper part and lower part of S-glycoprotein from SARS-CoV, while the less abundant high-mannose glycans are equally distributed along with the glycoprotein.
- in all three coronaviruses, the RBDs only contain complex N-glycans.

**Figure 28:**
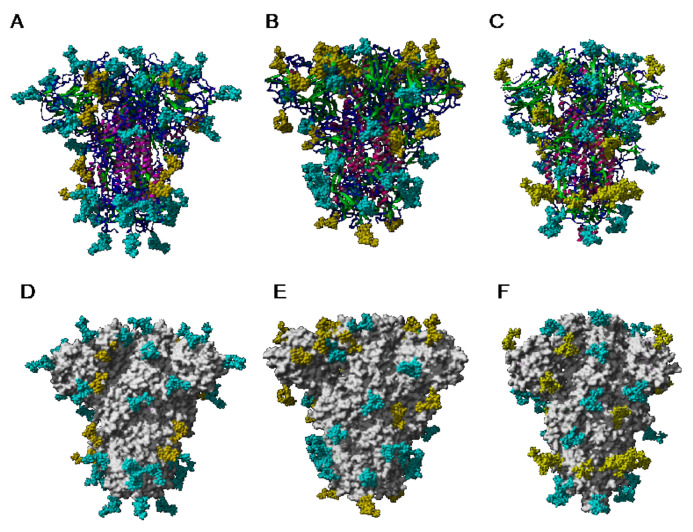
(**A**–**C**). Lateral face of the ribbon diagram representing the homotrimeric spike of SARS-CoV (PDB code 6ACD) (**A**), MERS-CoV (PDB code 5W9H) (**B**), and SARS-CoV-2 (PDB code 6VXX) (**C**). High-mannose glycans and complex glycans are colored yellow and cyan, respectively. (**D**–**F**). Lateral face of the molecular surface (colored grey) of the S-glycoprotein of SARS-CoV (**D**), MERS-CoV (**E**), and SARS-CoV-2 (**F**). High-mannose glycans and complex glycans are colored yellow and cyan, respectively.

**Figure 29:**
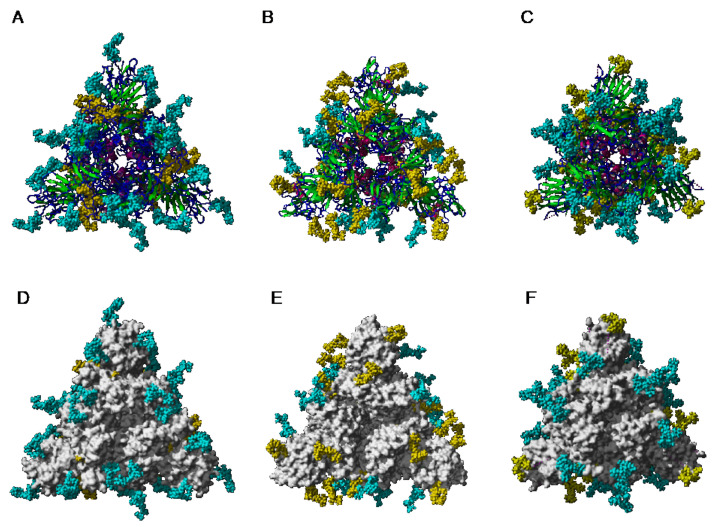
(**A**–**C**). Top face of the ribbon diagram representing the homotrimeric spike of SARS-CoV (PDB code 6ACD) (**A**), MERS-CoV (PDB code 5W9H) (**B**), and SARS-CoV-2 (PDB code 6VXX) (**C**). High-mannose glycans and complex glycans are colored yellow and cyan, respectively. (**D**–**F**). Top face of the molecular surface (colored grey) of the S-glycoprotein of SARS-CoV (**D**), MERS-CoV (**E**), and SARS-CoV-2 (**F**). High-mannose glycans and complex glycans are colored yellow and cyan, respectively.

According to the different distribution of high-mannose and complex N-glycans along the S-glycoprotein of SARS-CoV, MERS-CoV, and SARS-CoV-2, both types of glycans are differentially distributed at the surface of the spikes of SARS-CoV, MERS-CoV, and SARS-CoV-2, as shown from pictures of the lateral faces (Figure 28A–F) and front faces (Figure 29A–F) of the spikes:

Discrepancies observed in the distribution and exposure of both types of *N*-glycans, especially at the top of the spikes, between SARS-CoV, MERS-CoV, and SARS-CoV-2 (Figure 29), allow for predicting the quite different accessibility of the high-mannose and complex glycan shield of the different coronaviruses to the Man-specific lectins from plants, fungi, algae, and bacteria. 

**Figure 30:**
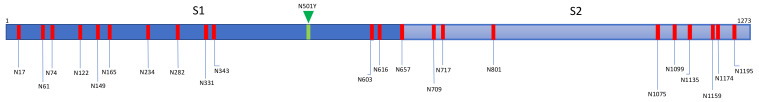
Localization of point mutation N501Y in the amino acid sequence of the S-glycoprotein from SARS-CoV-2. The actually glycosylated *N*-glycosylation sites distributed along the amino acid sequence are indicated by red rods and numbered.

Interestingly, none of the *N*-glycosylation sites was impacted by the point mutations identified on the SARS-CoV-2 variants B.1.351 and B.1.1.7 that display increased resistance to antibody neutralization [131]. The only asparagine residue impacted by the N501Y mutation in the U.K., South African, and Brazilian variants of SARS-CoV-2, is not part of an *N*-glycosylation site (Figure 30).

## 5. Man-Specific Lectins from Higher Plants, Fungi, Algae and Cyanobacteria, Specifically Interact with the Highly Glycosylated S Glycoprotein from Coronaviruses

**Figure 31:**
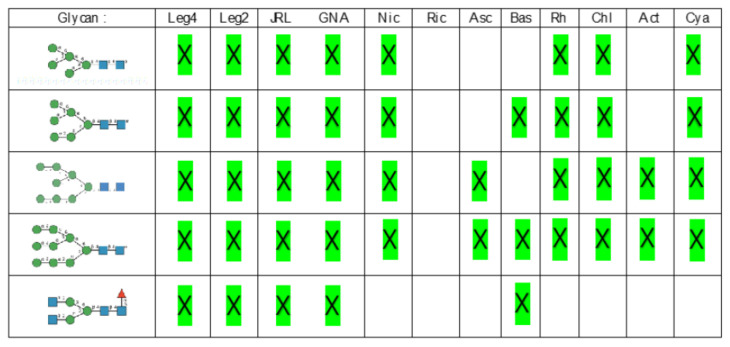
SARS-CoV glycans that can be recognized (X) by Man-specific lectins from plants, mushrooms, algae, and cyanobacteria, as predicted from the results of glycan arrays performed with purified lectins: Leg4 (Con A): single-chain legume lectins; Leg2 (PsA, LcA): two-chain legume lectins; JRL (Morniga-M): jacalin-related lectins; GNA (GNA, ASA): GNA-like lectins; Nic: Nictaba from *Nicotiana tabacum*; Ric: ricin-B from *Ricinus communis*; Asc (NcCVN): lectins from Ascomycota; Bas (ABA): lectins from Basidiomycota; Rh (griffithsin, KAA-2): lectins from Rhodophyta; Chl (BCA): lectins from Chlorophyta; Act (actinohivin): lectins from actinomycetes; Cya (cyanovirin): lectins from cyanobacteria.

**Figure 32:**
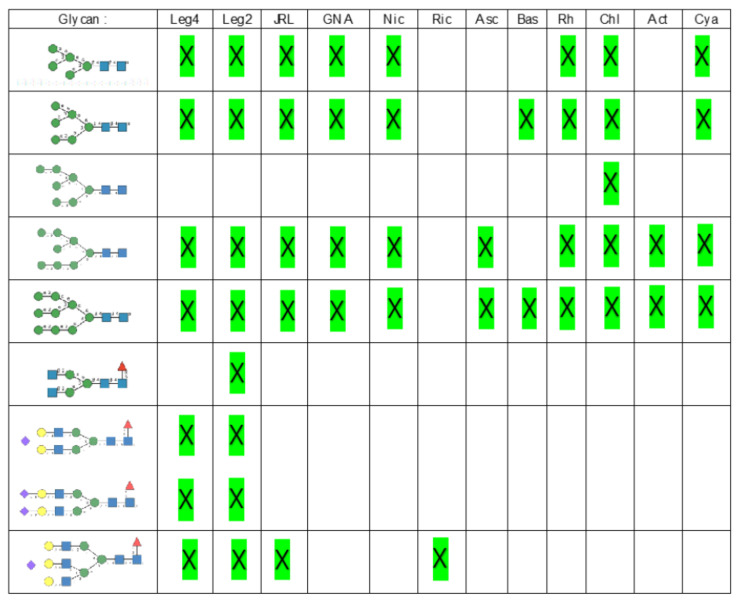
MERS-CoV glycans that can be recognized (X) by Man-specific lectins from plants, mushrooms, algae, and cyanobacteria, as predicted from the results of glycan arrays performed with purified lectins: Leg4 (Con A): single-chain legume lectins; Leg2 (PsA, LcA): two-chain legume lectins; JRL (Morniga-M): jacalin-related lectins; GNA (GNA, ASA): GNA-like lectins; Nic: Nictaba from *Nicotiana tabacum*; Ric: ricin-B from *Ricinus communis*; Asc (NcCVN): lectins from Ascomycota; Bas (ABA): lectins from Basidiomycota; Rh (griffithsin, KAA-2): lectins from Rhodophyta; Chl (BCA): lectins from Chlorophyta; Act (actinohivin): lectins from actinomycetes; Cya (cyanovirin): lectins from cyanobacteria.

**Figure 33:**
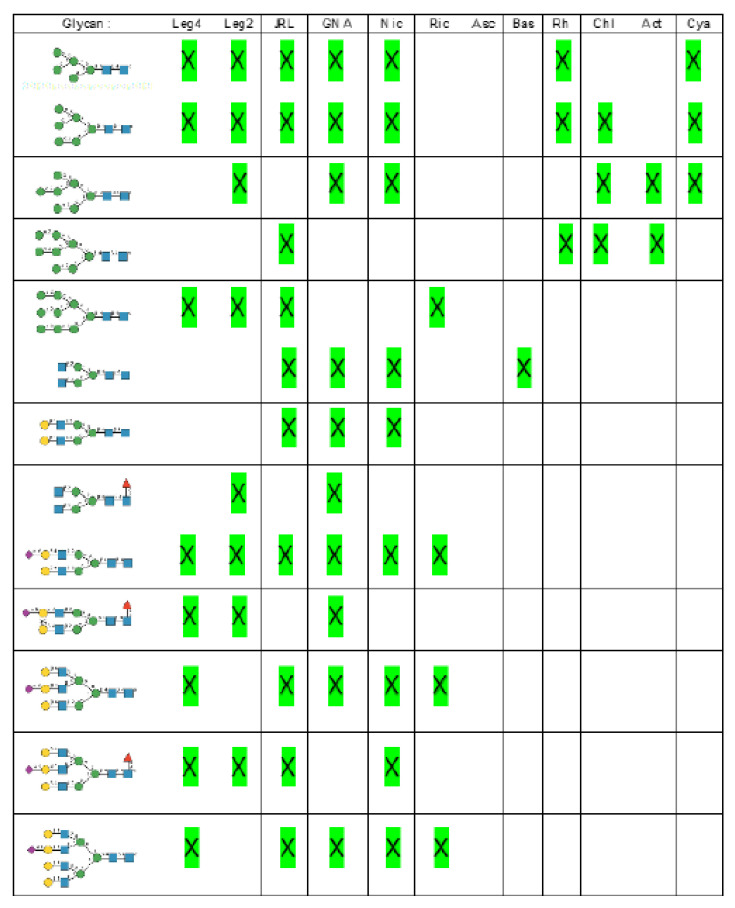
ARS-CoV-2 glycans that can be recognized (X) by Man-specific lectins from plants, mushrooms, algae, and cyanobacteria, as predicted from the results of glycan arrays performed with purified lectins: Leg4 (Con A): single-chain legume lectins; Leg2 (PsA, LcA): two-chain legume lectins; JRL (Morniga-M): jacalin-related lectins; GNA (GNA, ASA): GNA-like lectins; Nic: Nictaba from *Nicotiana tabacum*; Ric: ricin-B from *Ricinus communis*; Asc (NcCVN): lectins from Ascomycota; Bas (ABA): lectins from Basidiomycota; Rh (griffithsin, KAA-2): lectins from Rhodophyta; Chl (BCA): lectins from Chlorophyta; Act (actinohivin): lectins from actinomycetes; Cya (cyanovirin): lectins from cyanobacteria.

**Figure 34:**
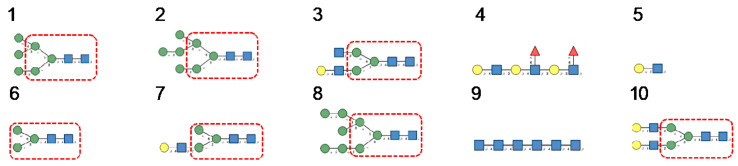
Glycans recognized by Nictaba in glycan array experiments, arranged from 1 to 10 in decreasing order of affinity.

Depending on the results obtained in glycan-binding assays and glycan array experiments, one can predict the potential interaction with high-mannose glycans and complex glycans identified in the glycan shield covering the spikes from SARS-CoV, MERS-CoV, and SARS-CoV-2 viruses (Figure 31, Figure 32 and Figure 33):

- Man-specific single- and two-chain lectins from the Fabaceae, which primarily recognize the (α3,α6)mannoside core of the branched GlcNAc_2_Man_3_ oligosaccharides, readily interact with the complex glycans and, to a lesser extent, with high-mannose glycans of the spikes from SARS-CoV (Figure 31), MERS-CoV (Figure 32) and SARS-CoV-2 (Figure 33). In this respect, two-chain lectins from the Viciae, e.g., pea lectin PsA and lentil lectin LcA, differ from single-chain lectins from other families of Fabaceae, e.g., Con A from *Canavalia ensiformis* or DGL from *Dioclea grandiflora*, by a much more pronounced affinity for α6-fucosylated complex glycans [21]. In addition, due to the particularly well-exposed distribution of complex glycans at the top of the SARS-CoV and SARS-CoV-2 spikes, both types of coronaviruses are predicted to better interact with lectins from the Fabaceae, compared to MERS-CoV spikes (Figure 29).
- The Man-specific jacalin-related lectins (JRL), such as Morniga-M from *Morus nigra*, which also recognizes *N*-glycans of the hybrid type in addition to complex glycans and high-mannose glycans, could serve as glycan probes for coronaviruses, particularly for binding to MERS-CoV, which exhibits hybrid glycans at the N1288 glycosylation site (Figure 26).
- The GNA-related lectins, which specifically interact with branched Man_5_-Man_9_ oligomannosides, would predominantly recognize high-mannose glycans (Figure 31, Figure 32 and Figure 33) and, especially, the well-exposed high-mannose glycans protruding at the top of the MERS-CoV spikes (Figure 29). The less exposed high-mannose glycans located at the bottom of the SARS-CoV and SARS-CoV-2 spikes (Figure 28), should also be recognized, but to a lesser extent.

- Nictaba lectin from *Nicotiana tabacum* interacts with the following glycans arranged in decreasing order of affinity according to glycan array experiments performed at the Consortium for Functional Glycomics, and should therefore recognize the complex glycans and high-mannose glycans forming the glycan shield of the spikes from coronaviruses (Figure 34):

- The ricin-B lectin from the *Iris hollandica* RIP-II was also shown to interact with complex (α6-fucosylated) glycans, high-mannose glycans, and hybrid glycans in glycan arrays experiments. Accordingly, it should readily interact with the spikes covering all types of coronaviruses (Figure 31, Figure 32 and Figure 33).
- Man-specific lectins from mushrooms belonging to the Ascomycota, essentially recognize high-mannose glycans (Nc-CVN from *Neurospora crassa*, and Tb-CVN from *Tuber borchii*, Flo1p from *Saccharomyces pasteurianus*, and Flo5A from *S. cerevisiae*), whereas other Man-specific lectins from Basidiomycota, e.g Abmb from *Agaricus bisporus*, and MOA from *Marasmius oreades*, preferentially recognize complex glycans. Mushroom lectins should recognize the high-mannose glycans and the complex glycans from coronaviruses.
- Man-specific lectins from red algae and green algae, both recognize exclusively high-mannose glycans containing Man_5_-Man_9_ mannose residues. They should be nicely adapted for the recognition of the high-mannose glycan shield exposed at the top of the MERS-CoV spikes (Figure 28 and Figure 29). Obviously, they should also recognize the high-mannose glycans associated with the SARS-CoV and SARS-CoV-2 spikes (Figure 31, Figure 32 and Figure 33).
- Man-specific lectins from algae, actinobacteria (actinohivin) and cyanobacteria (cyanovirin, microvirin, scytovirin), which predominantly possess a β−barrel structure and display an exclusive specificity for branched Man_5_-Man_9_ oligomannosides, should recognize the high-mannose glycan shield covering the coronavirus spikes and, especially, the MERS-CoV virus (Figure 28 and Figure 29).

The above predictions have already been corroborated by coronavirus-binding experiments using several lectins from plants, algae, and bacteria, confirming the potential of Man-specific lectins as relevant probes for the proper targeting of coronavirus spikes.

The pioneering work of van der Meer et al. (2007) demonstrated the anti-viral properties of Man-specific GNA-related lectins such as CA from *Cymbidium* sp., HHA from *Hippeastrum* hybrid, and GNA from *Galanthus nivalis* against SARS-CoV, and also highlighted the efficacy of the bacterial cyanovirin CVN [132]. These results were further extended to a broader list of 33 lectins, including Morniga-M from *Morus nigra* and other Man-specific lectins exhibiting a different structural scaffold [13]. The most efficient lectins against SARS-CoV predominantly consist of GNA-related Man-specific lectins (Table 1). In addition, lectins with different specificities, e.g., Gal-specific lectins (Morniga-G from *Morus nigra*), GalNAc-specific lectins (ML II from *Viscum album*), and GlcNAc-specific lectins (UDA from *Urtica dioica*), also displayed a significant anti-SARS-CoV activity. In addition, two targets for HHA, the Man-specific lectin from Amaryllis (*Hippeastrum* hybrid), were identified at the beginning and at the end of the infectious virus cycle. These target proteins most probably are involved in the viral attachment to the host cell surface and the virus release from the infected cells, respectively. The GNA-related Man-specific lectins interact with the glycan shield from the SARS-CoV spikes and thus interfere with the attachment of the virions to and the release from the host cells, with the exception of the garlic (*Allium sativum*) lectin ASA, which is devoid of any anti-viral activity against SARS-CoV. Obviously, the specific interaction of GNA-related lectins with the SARS-CoV virions depends on the preferential recognition by lectins of high-mannose glycans well exposed at the top of the SARS-CoV spikes (Figure 29).

**Figure 35:**
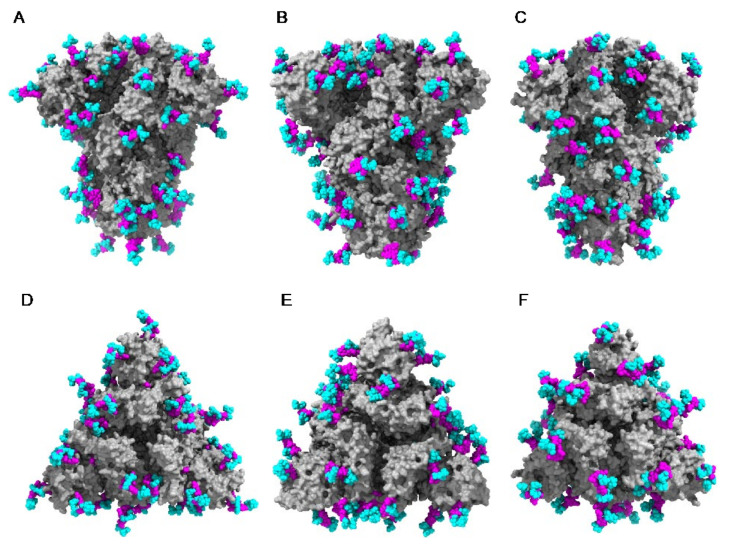
(**A**–**C**). Lateral views of the molecular surfaces (colored grey) of spikes from SARS-CoV (PDB code 6ACD) (**A**), MERS-CoV (PDB code 5W9H) (**B**), and SARS-CoV-2 (PDB code 6VXX) (**C**), showing the exposure of the fucosylated and non-fucosylated tri-mannosyl cores (colored magenta) of high mannose glycans and complex glycans (colored cyan). (**D**–**F**). Front views of the molecular surfaces (colored grey) from SARS-CoV (**D**), MERS-CoV (**E**), and SARS-CoV-2 (**F**), showing the exposure of the fucosylated or non-fucosylated tri-mannosyl cores (colored magenta) of high mannose glycans and complex glycans (colored cyan).

Recently, another Man-specific lectin FRIL from the legume *Lablab* (*Dolichos*) *purpureus* seeds, was shown to interfere with the SARS-CoV-2 entry in host cells, and its antiviral activity is mediated by specific recognition of complex glycans occurring on the surface of the S-glycoprotein [133]. In glycan array experiments, FRIL exhibited the best binding to complex-type N-glycans with α1,3 or α1,4 fucosylated sub-terminal GlcNAc whereas a slightly weaker binding was obtained with non-terminally fucosylated complex- and hybrid-type N-glycans. This situation is reminiscent of the preferential affinity of Man-specific Viciae lectins (LcA, LoL-I, PsA) for α1,6-fucosylated *N*-glycans of the complex type [21,134,135], with the difference that Viciae lectins bind α1,6-Fuc on reducing ends of complex-type N-glycans, which are therefore suspected to be less accessible to the lectins. However, Vicieae lectins also interact with the non-fucosylated tri-mannosyl core of high mannose and complex-type glycans, which appear well exposed on the surface of the coronavirus spikes (Figure 35A–F). This is consistent with the predicted preferential affinity of Man-specific legume lectins for α1,6-fucosylated *N*-glycans of the complex type which predominantly occur at the top of the SARS-CoV-2 spikes (Figure 29). These results obtained with a legume lectin, compared to those obtained with GNA-related lectins towards SARS-CoV, highlight the importance of a differential distribution of the high-mannose glycans and complex glycans on the surface of the spikes, which, in fact, define the type of glycans that will be accessible to the Man-specific lectins from the different groups.

Man-specific lectins from red algae, and especially griffithsin GRFT, were recognized very early as specific carbohydrate-binding agents for SARS-CoV, due to its strict specificity for high-mannose glycans containing Man_5_-Man_9_ units [136,137]. In addition, GRFT was identified as a potent inhibitor of the MERS-CoV infection, inhibiting the entry of virions into the cells [138]. Other griffithsin-related Man-specific lectins from Ascomycota, e.g., Nc-GRFT from *Neurospora crassa* and Tb-GRFT from *Tuber borchii*, should also interact with the glycan shield covering the SARS-CoV spikes. Actinohivin, microvirin, and scytovirin should also specifically interact with SARS-CoV but, to date, no information is available yet on their anti-SARS-CoV properties.

Similar to the Man-specific lectins from plants and algae, the so-called mannose-binding lectins MBL from animals and especially, the calcium-dependent serum C-lectins, which play a key role as opsonins in innate immunity towards a number of pathogens, including viruses, bacteria, and protozoa, were identified as selective inhibitors for the infection of host cells by SARS-CoV [139]. In addition, a single *N*-glycosylation site N330 of the SARS-CoV S-glycoprotein, predominantly occupied by *N*-glycans of the complex type (Figure 26), was identified as a critical target for the interaction from directed mutagenesis experiments. In contrast, other membrane-associated Man-specific lectins such as DC-SIGN and DC-SIGNR were assigned as receptors promoting infection and dissemination of SARS-CoV through the specific recognition of the glycan shield covering the virion spikes [140]. Recently, a detailed study of the interactions of the *N*-linked glycans in the RBD from the SARS-CoV-2 spike protein has been performed with human galectins, using NMR spectroscopy [141]. Moreover, the inhibition of Galectin-3, another animal lectin, was proposed as a possible treatment capable of impeding the SARS-CoV-2 attachment to the host cells and suppressing the host inflammatory response [142].

The mechanism by which lectins block viral replication once they have recognized the glycoproteins covering the enveloped viruses, still remains unclear.

Previous experiments performed to elucidate the inhibitory mode of action of Man-specific lectins (GNA from snowdrop *Galanthus nivalis*, and HHA from *Hippeastrum* hybrid) and UDA, the GlcNAc-binding lectin from stinging nettle *Urtica dioica*, on feline infectious peritonitis virus (FIPV) and mouse hepatitis virus (MHV), showed that lectins do not inhibit the attachment of coronavirus to the host cells but affect coronavirus entry at a post-binding stage. The lectins could interfere with some spike protein rearrangements required for driving the fusion between the viral envelope and the host cell membrane [143].

Griffithsin, the red algal Man-specific lectin, was identified as a potent inhibitor of MERS-CoV infectivity and replication in vitro. The lectin was shown to interact with the MERS-CoV spike protein during the early attachment step to the virions, suggesting that griffithsin could prevent the attachment of the virions to the DPP4 receptors of the host cells. Accordingly, the lectin cannot block MERS-CoV infection when added to the virions at a post-binding step [138].

Investigating the mechanism by which FRIL neutralizes influenza viruses H1N1, H3N2, H5N1, and H7N9, under in vitro and in vivo conditions, Liu et al. (2020) showed that (1) FRIL neutralizes only influenza viruses possessing complex-type *N*-glycans, (2) due to its tetravalent character, FRIL binds to the virions and creates virion aggregates before the endocytosis into the host cell, (3) FRIL halts the host cell infection process in the late endosome/lysosome and thus prevents the nuclear import of the virions [133]. According to these results, the authors proposed a model in which the lectin causes the virion aggregation outside the host cell and, once endocytosed, prevents their nuclear import by entrapping the virion-lectin aggregates in the late endosome/lysosome stage. In this process, the multivalency of FRIL appears as a key factor for bridging the influenza virions and create virion aggregates outside the host cell, prior to their endocytosis. Similarly, FRIL also binds to the complex-type glycans of SARS-CoV-2 virions and displays a lower affinity towards high-mannose glycans. These results confirm that FRIL exhibits a better affinity to the complex-type glycans with α1,3 or α1,4 fucosylated sub-terminal GlcNAc, compared to α1,3 and α1,6 linked Man of the α1,6-fucosylated or non-fucosylated tri-mannosyl core GlcNAc_2_Man_3_. The affinity of FRIL for complex-type glycans could explain why the lectin neutralizes both influenza and SARS-CoV-2 viruses but has almost no effect on HIV.

## 6. Biomedical Perspectives

**Figure 36:**
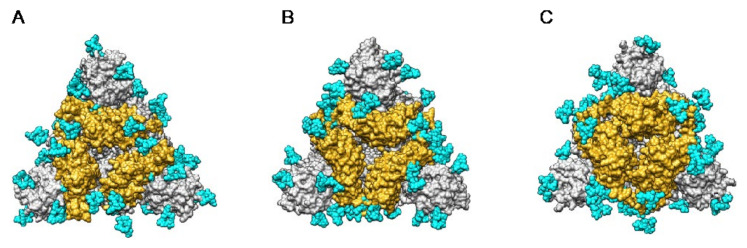
(**A**–**C**). Top face of the ribbon diagram of the homotrimeric spike of SARS-CoV (PDB code 6ACD) (**A**), MERS-CoV (PDB code 5W9H) (**B**), and SARS-CoV-2 (PDB code 6VXX) (**C**). High-mannose glycans and complex glycans are colored cyan. Molecular surfaces corresponding to the RBDs are colored gold and the remaining molecular surfaces are colored grey.

The extensive glycosylation at the surface of the S protein offers a shielding effect to SARS-CoV, MERS-CoV, and SARS-CoV-2. In addition to this protective role, a possible stabilization function has been postulated for some of the *N*-glycans covering the SARS-CoV-2 S protein, that could be exploited in the future for targeting the glycan coat by therapeutic drugs [144]. However, the distribution of the glycan shield at the surface of the spikes and especially, at the top of the spikes, does not consistently support a protective function susceptible to reduce the vulnerability of the coronaviruses towards antibody epitopes and/or exogenous proteases. In this respect, the exposed area of the RBDs at the top of the spikes is substantially free of glycans and thus, remains available for specific recognition by the opened spike conformation of the corresponding ACE2 (SARS-CoV and SARS-CoV-2) and DPP4 (MERS-CoV) host cell receptors (Figure 36A–C).

**Figure 37:**
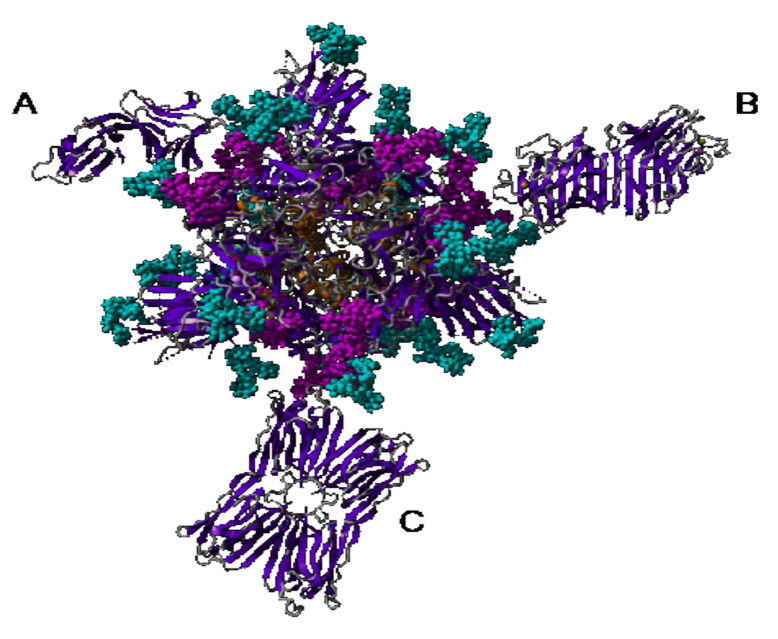
Cartoon showing the relative size of different Man-binding lectins (dimeric GNA-like lectin (**A**), bivalent legume lectin β_2_α_2_ (**B**), and Jacalin-related lectin (**C**)), complexed to the homotrimeric spike (front face) from SARS-CoV-2.

Nevertheless, the steric hindrance created by the interaction of Man-specific lectins such as the dimeric PsA (50 kDa), the tetrameric GNA (50 kDa), and Morniga-M (60 kDa) or the octameric Heltuba (120 kDa), with the high-mannose and complex *N*-glycans located in the vicinity of the RBD area, should interfere with the receptor recognition process and could disturb or prevent the entry of coronaviruses in host cells. In this respect, the di-, tetra-, hexa- (*Parkia platicephala* lectin), and octameric Man-specific lectins from higher plants, which exhibit high molecular weights in the range between 50–100 kDa, could serve as blocking agents that can prevent the coronavirus infection. Other Man-specific lectins from algae, fungi, and cyanobacteria seem to be less adapted to act as a blocking agent owing to their smaller size, e.g., 25 kDa for the griffithsin swapped homodimer and 20 kDa for the cyanovirin swapped homodimer, respectively (Figure 37).

As blocking agents for coronaviruses SARS-CoV, MERS-CoV, and SARS-CoV-2, Man-specific lectins from higher plants, algae, fungi, and cyanobacteria could be used to entrap and immobilize the virions using air-conditioned filters impregnated with lectins to prevent the coronavirus dissemination by contaminated people. However, the covalent fixation of lectins to air-conditioned filters necessitates large amounts of purified lectins, which may be problematic in view of low yields usually obtained during purification of Man-specific lectins from higher plants. Recent improvements performed in the large-scale purification of the algal lectins from *Eucheuma* [114], and griffithsin from tobacco leaves, could provide the bulk production of Man-specific lectins required for the manufacturing of lectin-impregnated filters [145]. However, the blocking activity of dried lectins immobilized on air-conditioned filters seems unlikely because these proteins need water to retain both their conformation and carbohydrate-binding activity. At most, one can imagine a filtration device equipped with a wet filter containing immobilized lectins, which would remove the air from the virions before entering the air-conditioned filter. 

Another potential biomedical application for Man-specific lectins could consist of their use as glycan probes for the detection of coronaviruses in the environment, e.g., on various domestic surfaces such as doorknobs, home furnishings, handrails, mirrors, computers, etc. Virions deposited on these surfaces could be detected under UV illumination, e.g., using specific antibodies labeled with a fluorochrome.

It is possible to imagine further therapeutic issues for Man-specific lectins with respect to the coronavirus infection. Recently, a risk of contamination by SARS-CoV-2 has been identified via spoiled blood or blood products transfused to healthy people [146]. Although the risk of a transfusion transmission is essentially theoretical [147], a plasmapheresis through a column of immobilized Man-specific lectin would be sufficient to eliminate the eventual blood contaminants. A similar filtration of the suspected blood samples through a column containing an immobilized Man-specific lectin could be performed in blood centers or blood banks and would be a control measure sufficient to purge the blood from coronavirus particles and thus prevent possible contamination for the transfused people. In this respect, up to 80% of MERS-CoV and 70% of MARV (Marburg virus), were successfully eliminated by lectin affinity plasmapheresis using GNA as a blocking agent immobilized in the extra-capillary space of a standard plasma filter [148].

From the scarce in vitro experiments conducted to study the effects of Man-specific lectins from higher plants [13,132,133], and griffithsin [137,138], towards MERS-CoV and SARS-CoV-2, the anti-viral activity of lectins appeared to prevent both attachment and entry of the virus in the host cell but does not interfere with the coronavirus replication process within the cell. Consequently, Man-specific lectins cannot be considered as replication blockers for coronaviruses. A very different situation occurs for other enveloped viruses such as HIV-1 [24], influenza virus [37], and Herpes virus [25], whose replication is apparently blocked in vitro, in the presence of Man-specific lectins. 

In addition, some of the biomedical applications presented here could be adapted to veterinary medicine by addressing animal coronavirus concerns [149] as part of the “one health” approach.

## 7. Bioinformatics

The X-ray coordinates available for lectins, lectins in complex with simple sugars, oligosaccharides, and more complex glycans, and S-glycoproteins from SARS-CoV, MERS-CoV, and SARS-CoV-2, were taken from the PDB.

Homology modeling of other lectins was performed with the YASARA Structure program [150] using various structurally related proteins as templates from the PDB [151], depending on the overall structural scaffold to which they belong. PROCHECK [152], ANOLEA [153], and the calculated QMEAN scores [154,155] were used to assess the geometric and thermodynamic qualities of the resulting three-dimensional models.

Docking of simple sugars and oligosaccharides was performed with YASARA and SwissDock [156,157]. The molecular surface of the lectins and S-glycoproteins from SARS-CoV, MERS-CoV, and SARS-CoV-2, were calculated and displayed with Chimera [158]. Molecular cartoons were drawn with Chimera and YASARA.

Assuming that putative *N*-glycosylation sites Asn-X-Thr/Ser of SARS-CoV, MERS-CoV, and SARS-CoV2, are actually glycosylated, a classic bi-antennary high-mannose glycan chain with a tri-mannoside core (Man)_2_-(Man)_3_-(GlcNAc)_2_ was modeled using the GlyProt server (http://www.glycosciences.de/modeling/glyprot/php/main.php, accessed on 1 March 2021) [159], and represented in CPK on the molecular surface of the spike S-glycoproteins. According to the known differential distribution of high-mannose *N*-glycans and complex *N*-glycans on the surface of the S-glycoproteins, another run of modeling was performed using standard high-mannose glycans and complex glycans as models.

Cartoons for high-mannose glycans, complex *N*-glycans, and hybrid glycans, were built and represented with the DrawGlycan SNFG package for Mac [160]. Classical colored symbols were used for representing Fuc (red triangle), Gal (yellow circle), GalNAc (yellow square), GlcNAc (blue square), Man (green circle), and sialic acid/Neu5Ac (purple losange), respectively.

## 8. Discussion

Glycans of the S-glycoproteins forming the spikes of SARS-CoV, MERS-CoV and SARS-CoV-2, consist of high-mannose glycans and often sialylated *N*-glycans that predominantly occupy their *N*-glycosylation sites [11,12]. However, depending on the coronaviruses, some discrepancies occur between the distribution of the two types of glycans on the surface of the virion, which introduces some diversity in the glycan shield covering the coronavirus spikes [11]. Accordingly, Man-specific lectins from plants, algae, fungi, and bacteria, which differ slightly due to their fine sugar-binding specificities, offer a vast panel of glycan probes more or less adapted to the specific recognition of the different coronaviruses. In this respect, GNA-related lectins together with Man-specific lectins from algae and cyanobacteria, appear as glycan probes nicely adapted to the recognition of the high-mannose shield which predominates at the top of the MERS-CoV spike. Otherwise, legume lectins with a higher affinity for *N*-glycans possessing the trimannoside Manα1,3Manα1,6Man core, seem better adapted to the recognition of the *N*-glycans distributed predominantly at the top of the glycan shield from SARS-CoV and SARS-CoV-2.

Besides Man-specific lectins, other lectins displaying different carbohydrate-binding specificities have previously been identified as glycan probes for MERS-CoV [13,161]. In this respect, lectins exhibiting Man/Glc-specificity (CLA from *Cladrastris lutea*), Gal/GalNAc-specificity (Morniga-G from *Morus nigra*), GlcNAc-specificity (Nictaba from *Nicotiana tabacum*), chitin-specificity (UDA from *Urtica dioica*), display an antiviral activity often higher or similar to those displayed by genuine Man-specific lectins, essentially the GNA-related lectins from monocot plants [39]. The occurrence of (sialylated)complex glycans and hybrid glycans arrayed on the surface of coronavirus spikes, readily accounts for the differences observed among the carbohydrate-binding specificities towards the coronaviruses. Inhibition of the SARS-CoV replication was observed in a lethal SARS-CoV-infected BALB/c mouse model after treatment with the (GlcNAc)_n_-binding stinging nettle (*Urtica dioica*) lectin [161]. Nictaba from *Nicotiana tabacum* and UDA, two GlcNAc-binding lectins, also displayed antiviral properties against several families of enveloped viruses including influenza A/B, Dengue virus type 2, herpes simplex virus, and HIV, respectively [162]. Accordingly, a mixture of lectins with different carbohydrate-binding specificities should provide a glycan probe tool well adapted to all of the coronavirus types and strains.

## Figures and Tables

**Table 1:** Overview of Man-specific lectins from higher plants investigated in [13], for their anti-SARS-CoV activity, classified in decreasing order of activity (EC50 (μg/mL)). Adapted from [13].

Lectin Family	Plant Species	Lectin	Structural Scaffold	EC50 (μg/mL)
GNA-related	*Allium porum*	APA	β-trefoil	0.45 ± 0.08
	*Epipactis helleborine*	EHA	β-trefoil	1.8 ± 0.3
	*Listera ovata*	LOA	β-trefoil	2.2 ± 1.3
	*Hyppeastrum hybrid*	HHA	β-trefoil	3.2 ± 2.8
	*Cymbidium hybrid*	CA	β-trefoil	4.9 ± 0.8
	*Narcissus pseudonarcissus*	NPA	β-trefoil	5.7 ± 4.4
	*Galanthus nivalis*	GNA	β-trefoil	6.2 ± 0.6
	*Allium ursinum*	AUA	β-trefoil	18 ± 4
	*Tulipa hybrid*	TL MI	β-trefoil	22 ± 6
	*Lycoris radiata*	LRA	β-trefoil	48
	*Colocasia esculenta*	CEA	β-trefoil	>60
	*Allium sativum*	ASA	β-trefoil	>100
JRL-related	*Morus nigra*	Morniga-M	β-barrel	1.6 ± 0.5
Nictaba-related	*Nicotiana tabacum*	Nictaba	β-sandwich	1.7 ± 0.3
Legume lectins	*Cladrastis lutea*	Cladrastis	β-sandwich	7.4 ±0.2
